# Genetic Predisposition to Pass the Standard SICCT Test for Bovine Tuberculosis in British Cattle

**DOI:** 10.1371/journal.pone.0058245

**Published:** 2013-03-06

**Authors:** William Amos, Ellen Brooks-Pollock, Ruth Blackwell, Erin Driscoll, Martha Nelson-Flower, Andrew J. K. Conlan

**Affiliations:** 1 Department of Zoology, Cambridge University, Cambridge, Cambridgeshire, United Kingdom; 2 Department of Veterinary Medicine Disease Dynamics Unit, Cambridge University, Cambridge, Cambridgeshire, United Kingdom; 3 Animal Health and Veterinary Laboratories Agency (AHVLA), New Haw, Addlestone, Surrey, United Kingdom; University of Ottawa, Canada

## Abstract

Bovine tuberculosis (bTB) imposes an important financial burden on the British cattle industry, yet despite intense efforts to control its spread, incidence is currently rising. Surveillance for bTB is based on a skin test that measures an immunological response to tuberculin. Cattle that fail the test are classified as “reactors” and slaughtered. Recent studies have identified genetic markers associated with the reaction of cattle to the tuberculin test. At marker INRA111 a relatively common ‘22’ genotype occurs significantly more frequently in non-reactor cattle. Here we test the possibility that the putative protective ‘22’ genotype does not confer resistance but instead causes cattle that carry it to react less strongly to the prescribed test, and hence avoid slaughter, potentially even though they are infected. We show that, after controlling for age and breed, ‘22’ cattle react less strongly to the immunological challenge and may therefore be less likely to be classified as a reactor. These results highlight the potential discrepancy between infection and test status and imply that the effectiveness of the test-and-slaughter policy may be being compromised by selection for cattle that are genetically predisposed to react less strongly to tuberculin.

## Introduction

Bovine tuberculosis (bTB), caused by *Mycobacterium bovis*, is a chronic infectious disease that in the British cattle herd costs at least an estimated £91 million per year [1]. Despite extensive control efforts through a national test and slaughter programme, there has been a relentless increase in incidence over the past 20 years [2]. The factors driving this epidemic are still poorly quantified, in particular with respect to the relative contributions of cattle-to-cattle transmission [3], infection missed by testing [4] and wildlife reservoirs of infection [5]. In recent years, considerable scientific, popular and political attention has been focused on the role played by badgers and the efficacy of culling [6]–[8]. However, there is still considerable uncertainty about the basic natural history of infection in cattle in particular the genetic determinants of susceptibility and the relationship between infection status and reaction to standard diagnostic tests. In humans and wild boar, genetic factors have been linked to TB susceptibility [9]–[11]. Similar patterns may be expected in cattle [12] and heritable variability in susceptibility has been observed [13], with recent studies identifying associations between specific genotypes and the probability of being identified as being infected [14], [15]. Here we extend previous results and quantify the relationship between genetic marker INRA111 and measurements associated with the tuberculin skin test for bTB.

In Great Britain (GB) the standard screening test for bTB is the single intradermal comparative cervical tuberculin (SICCT) test [16], . The SICCT test compares immune responses to intradermal injections of bovine and avian tuberculin, purified protein derivatives (PPD) from *M. bovis* (bovine) and *M. avium* (avian) respectively. By measuring the difference in reaction between bovine and avian tuberculins, the test seeks to control for exposure to non-pathogenic mycobacteria [16], [17]. Threshold conditions are based jointly on a minimum swelling size at the bovine injection site and a minimum excess swelling size at the bovine compared with the avian injection site. Cattle who exceed the prescribed thresholds are classified as ‘reactors’ and are slaughtered. Herds in which reactors are identified are then subject to the imposition of movement restrictions and additional testing [4].

SICCT testing has been used routinely within GB since the introduction of a national test-and-slaughter programme in 1950 [2]. This programme led to the effective eradication of bTB within the GB herd by 1960, leading to subsequent reductions in the frequency of testing. However, since 1980 there has been a growing epidemic in the face of intensified testing efforts [2]. No clear single factor can be attributed to driving this rise in incidence that coincided with several changes in surveillance and husbandry [18] including: relaxation in the intensity of testing since 1993 [2], a shift from traditional British breeds to a higher proportion of higher yielding breeds [16], a reduction in the number of herds and increase in the average herd size [3], the change from using human to bovine tuberculin for testing [2] and suspected changes in epidemiology of bTB within the wildlife reservoir, in particular badgers [5].

Given the intensity of the test-and-slaughter policy and the fact that there is likely to be heritable variability in resistance/susceptibility, one might expect the British herd to show an increase in the frequency of genotypes that confer natural resistance to infection. That prevalence of bTB in the British herd remains high could indicate one of several scenarios. First, genetic factors may be negligible relative to other risk factors. Second, local selection for resistance/reduced susceptibility in regions of high bTB prevalence might be mitigated by herd structure, with many cattle fathered by artificial insemination using stud bulls raised elsewhere in the country. Third, genetic variability for resistance might be either non-heritable, perhaps because it operates through heterozygote advantage, or has already been eliminated. One further possibility relates to the proximate selection imposed by the test-and-slaughter policy that acts on ability to pass the test much more than the indirect target of disease susceptibility. Logically, selection will act on any heritable variation that, for a given degree of infection, causes a better chance of passing the test. For example, cattle that are hyper-sensitive to the avian challenge might produce larger avian swellings, reducing the bovine-avian swelling size differential. Equally, cattle with a reduced overall cell-mediated immune response will produce smaller swellings at both injection sites and again be more likely to pass.

We have previously identified two candidate markers that, in a modest sample of 384 cattle of mixed breeds, appear to be significantly associated with whether or not an animal at slaughter is classified as a reactor or a non-reactor [14]. Of these, the weaker effect is linked to a microsatellite lying near to IFNGR1, a well-known component of the immune system that has been linked directly to susceptibility to infection by bacterial diseases including tuberculosis [19]. In contrast, the stronger effect involves locus INRA111, a microsatellite that does not lie close to a gene associated with susceptibility. INRA111 was originally included because it showed a weak association with footrot in sheep and the nearest gene is vaccinia related kinase 2 (VRK2), a gene linked to the inflammatory response through an interaction with interleukin-1B [20].

One specific INRA111 genotype, the ‘2’ allele homozygote (‘22’), varies considerably in frequency between breeds and appears protective from the disease, being significantly enriched among non-reactors in a number of common breeds [14]. If the ‘22’ genotype is genuinely protective, an intense test-and-slaughter policy should select for an increase in frequency of the ‘2’ allele. That the ‘2’ allele has not been fixed might indicate one of several possible scenarios. Simplest would be that there has been insufficient time or selection pressure for large-scale changes in frequency to occur. Another possibility arises from the fact that INRA111 is a presumed neutral marker that only shows an association with reactor status indirectly through being in linkage disequilibrium (LD) with a neighbouring gene. LD is never perfect, and it is not impossible that the ‘22’ homozygote is actually marking a protective heterozygote at the gene itself. A third possibility is that ‘22’ animals are not resistant at all, but instead merely have characteristics that make them more likely to pass the SICCT test. Here we expand our sample set and test this hypothesis by exploring the relationship between the ‘22’ genotype and skin thickness measurements collected during routine SICCT testing. We find evidence of smaller skin test measurements for ‘22’ cattle, making them more likely to pass the test and hence to remain in the herd regardless of whether they are infected.

## Methods

### Samples and Genotyping

Tissue samples were collected opportunistically with permission at two abattoirs in southwest England, Ensors in the Forest of Dean and Jarrett’s in Bristol, each being a circle of skin surrounding an ear tag preserved in 96% ethanol. Our study began with 543 samples collected from Ensors by Erin Driscoll and included 44 different breeds, being reduced to 384 comprising the 10 best-represented breeds for the initial analysis. Subsequent reactor cattle were sampled at Ensors by the Meat Hygiene Service who kindly passed sub-samples to us. For a different geographic view and to augment our sample of non-reactor dairy cattle, further samples were collected during 2011 from Jarrett’s abattoir, Bristol. The final sample set comprised 1810 animals, including the 542 genotyped previously. The new animals were genotyped for INRA111 following existing protocols [14].

### National Testing Data

As a notifiable disease in Great Britain, national testing data on bTB are routinely collected by the Animal Health and Veterinary Laboratories Agency (AHVLA) on behalf of the Department of Environment, Food and Rural Affairs (DEFRA). SICCT testing in GB is conducted, and interpreted, according to the protocol set out at Annex B of EU Directive 64/432/EEC [21]. Briefly, avian and bovine tuberculin is injected into the cervical region and four skin thickness measurements are taken using callipers. The first pair of measurements corresponds to the measured skin thicknesses at the avian (*a1*) and bovine (*b1*) sites before injection of tuberculin. After 72 hours the skin thickness is measured again at the same sites giving the second pair of measurements (*a2*, *b2*). All measurements are rounded to the nearest mm and diagnostic status is determined by the difference of the differences: (*b2–b1*) – (*a2–a1*). Animals with a difference between avian and bovine reactions of at least 4 mm are classified as reactors under the standard interpretation of the test. Smaller differences of at least 2 mm are required under the severe interpretation of the test used when there is additional evidence of infection in the herd. Finally, a difference of 1–3 mm leads to an inconclusive reactor (IR) classification and retesting after 60 days. If an IR animal is still IR when retested it is reclassified as a reactor.

Swelling size measurements are recorded in both VetNet and the online VeBus database [7]. VeBus holds the individual measurements (a1, a2, b1 and b2) from all tests (failed or not) for a subset of animals, while VetNet holds data for the last test only of all cattle classified as reactors. Two important biases affect these measurements: (1) all reactors have, by definition, exceeded the prescribed threshold, so the expectation of any relationship with ‘22’ is unclear; (2) repeat testing can potentially lead to smaller swellings [22]. Consequently, for both consistency and to control for desensitisation we used the first non-reacting test results for each animal recorded in VeBus, regardless of whether that animal went on to become a reactor later in its life. We identified 573 individual cattle that were both genotyped for locus INRA111 and recorded in VeBus. For each of these we extracted age at testing and breed classification from the Cattle Tracing System (CTS). Under-represented breeds with fewer than 5 samples were dropped from the analysis leaving a final sample size of 526 (Table 1).

**Table 1:** Sample set used in the current study.

Code	Full Name	O-NR	O-R	N-NR	N-R
AA	Aberdeen Angus	4	1	7	12
AAX	Aberdeen Angus X	10	2	6	19
BAX	Blonde d’Aquitaine X	3	4	1	
BBX	Belgian Blue X	3	4	7	7
BF	British Friesian			11	30
BFX	British Friesian X			9	10
CH	Charolais			1	8
CHX	Charolais X	18	8		10
DEV	Devon			3	2
DEX	Dexter				5
FR	Friesian			10	11
HE	Hereford			11	8
HEX	Hereford X	12	2	13	7
HF	Holstein Freisian	3	11	25	41
HFX	Holstein Friesian X			2	4
HO	Holstein		2	7	8
J	Jersey			1	9
LIM	Limousin			6	
LIMX	Limousin X	35	5	8	22
SD	South Devon			4	9
SMX	Simmental X	11	3	2	17
WB	Welsh Black			12	

These represent 625 animals and 22 breeds drawn from an original set of 1810 samples collected. Qualifications for inclusion are: being genotyped for marker INRA111, having passed their first SICCT test, being recorded in the VeBus database and having at least four other samples from the same breed. The Table lists, for each of these breeds, the abbreviated code, the full breed name and the numbers of non-reactors (NR) and reactors (R), partitioned by whether they derived from the original (O-) Driscoll et al. study [14] (n = 141) or are new (N-) samples (n = 385).

All statistical analyses were conducted using the R statistical software package [23]. Logistic regression models were fitted using the glm() function and the significance of regression parameters was calculated using likelihood ratio tests by the standard glm methods within R. Stepwise model selection was carried out to select the most parsimonious model supported by the data using the stepAIC function of the “MASS” package [24]. Zero-inflated count regression models were fitted using “zeroinfl” [25] from the “pscl” package [26]. Bootstrapped confidence intervals were calculated using the boot package [27], [28] and goodness-of-fit of logistic regression models was assessed using functions from the “binomTools” package.

## Results

**Figure 1:**
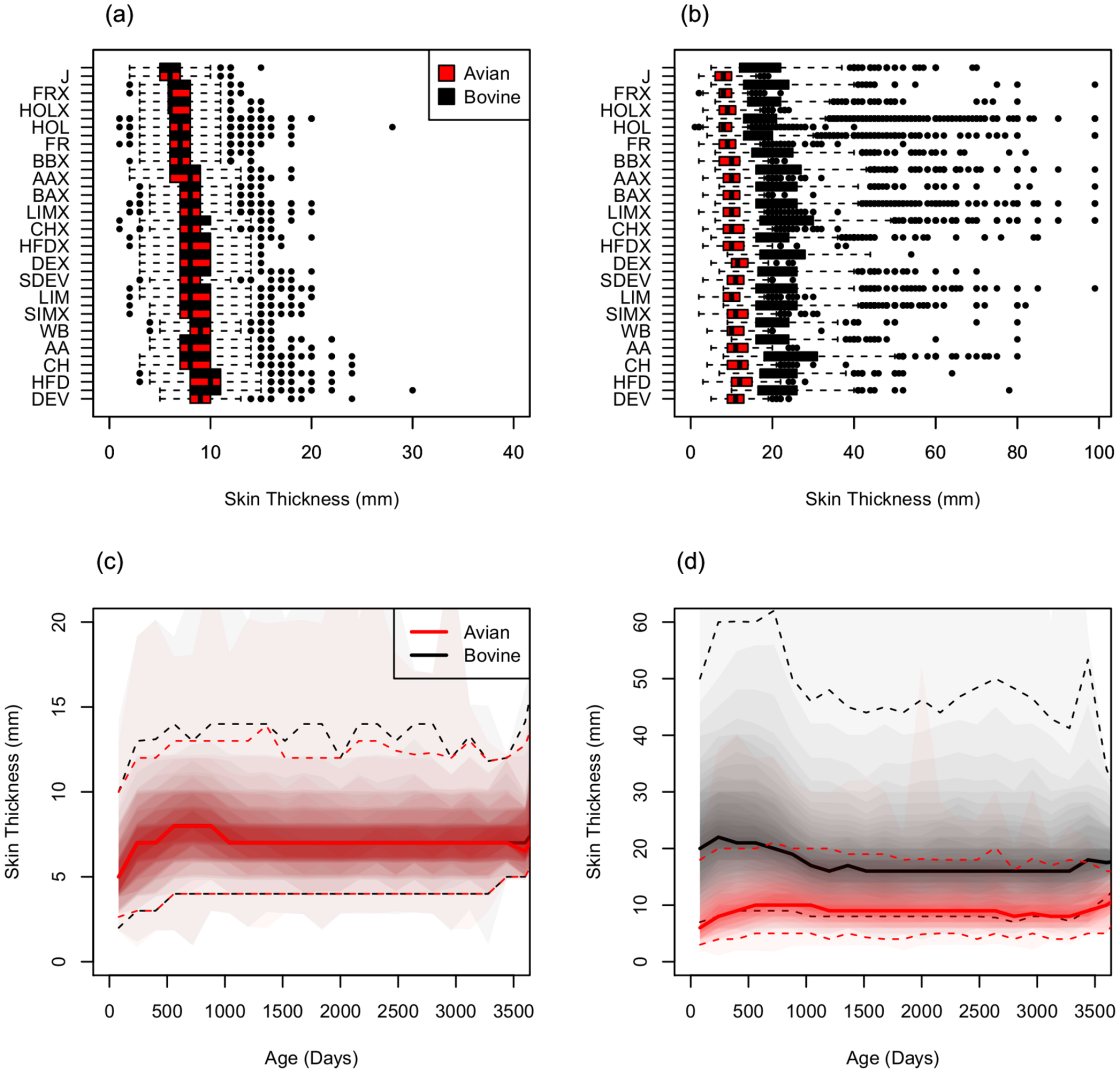
Recorded skin size measurements for reactor animals in VeBus. (**a**) Distribution of first avian (a1) and bovine (b1) measurements stratified by breed for all reactor animals recorded with VeBus and ordered by increasing median value; (**b**) Distribution of second avian (*a2*) and bovine (*b2*) measurements stratified by breed for all reactor animals recorded with VeBus; (**c**) Age profile of first avian (*a1*) and bovine (*b1*) measurements for all reactor animals in VeBus; (**d**) Age profile of second avian (*a2*) and bovine (*b2*) measurements for all reactor animals in VeBus. Given the variability in recorded measurements summary measures are potentially misleading. To demonstrate this variability we plot age profiles as a shaded density strip where the intensity of shading is proportional to the probability of a value at that point [44]. Median (solid lines) and upper and lower 95% ranges (dashed lines) are indicated.

The magnitudes of skin test measurements differ between breeds and with respect to age (Figure 1). A range of factors, immunological, epidemiological and physiological are likely to be contribute to these patterns. Cumulative exposure to *M. bovis* and other *Mycobacterium* spp. will increase with age. The immunological response to challenge is also likely to vary with host age and exposure to tuberculin through repeated testing [9]. In order to attempt to control for some of this variation in exposure and test history we extracted each animal’s first test recorded in VeBus and tested for an association with ‘22’ status, recorded as a binary character (‘22’ = 1, any other genotype = 0).

Baseline skin thickness for cattle (*a1*, *b1*) is known to vary between breeds [29] and to increase with age [30]. For the selected breeds in our study population, average skin thickness before inoculation recorded in VeBus ranges from 5.59 mm in Jersey cattle (J) to 8.05 mm in Welsh Black (WB) (*a1*, Table S1). This variation in skin thickness is accounted for in the SICCT test interpretation by subtracting the baseline measurement (*a1*, *b1*) from the final measurement (*a2*, *b2*). For the majority of animals recorded in VeBus the recorded differences (*da* = *a2*−*a1*, *db* = *b2−b1*) in non-reactors are zero (94.5% of avian measurements and 97.2% of bovine measurements). Observational biases in the recording of skin test thickness may be coloured by post-hoc interpretation of the test, with reactor animals receiving more careful measurement. Compliance of testers in GB is not systematically audited, so the relative biases introduced into each of the skin test measurements cannot be quantified. Consequently, we look for associations between genotype and all of the individual skin test measurements (*a1*, *b1*, *a2*, *b2*) in addition to the differences (*da*, *db*) required for diagnosis.

We first fitted a logistic regression model with ‘22’ status as a binary response and age, breed, *a1*, *b1*, *a2*, *b2*, *da*, *db* as predictors to test for associations between skin test measurements and genotype. Following a bi-directional step-wise AIC procedure to select the most parsimonious model, age, breed, *da*, *db* and *b1* were retained in the final model. No evidence for a lack of fit was found using Hosmer and Lemeshow’s goodness-of-fit test (p = 0.51) [31]. Predictive ability of the final model was assessed through the receiver operating characteristic (ROC) curve, with an area under the curve (AUC) of 0.68 [31]. The selected model suggests a significant association (p = 0.04) of ‘22’ status with the avian difference (da) after accounting for age and breed (Table 2).

**Table 2:** Test and animal factors associated with probability of being the ‘22’ genotype.

	Odds Ratio (95% CI)	z value	Pr(>|z|)
(Intercept)	1.8 (0.97–3.5)	1.87	0.06
Age	1.0 (1.0–1.0)	1.52	0.13
***da***	**0.76 (0.57–0.96)**	−**2.06**	**0.039**
*db*	1.3 (0.93–1.99)	1.40	0.16
*b1*	0.91 (0.83–1.00)	−1.84	0.066
**AA**	**7.7 (2.3–37)**	**2.97**	**0.003**
AAX	1.6 (0.72–3.6)	1.13	0.26
BAX	0.46 (0.09–2.0)	−1.01	0.31
BBX	0.57 (0.20–1.6)	−1.08	0.28
CH	0.40 (0.080–1.6)	−1.21	0.23
CHX	1.7 (0.75–4.0)	1.25	0.20
DEV	1.7 (0.25–14)	0.53	0.60
DEX	0.20 (0.098–1.50)	−1.40	0.16
FR	1.2 (0.62–2.3)	0.52	0.60
FRX	1.4 (0.51–4.10)	0.64	0.52
HFD	0.47 (0.15–1.4)	−1.32	0.19
HFDX	0.58 (0.26–1.3)	−1.36	0.17
HOLX	2.9 (0.42–60.0)	0.96	0.34
J	0.54 (0.13–2.0)	−0.89	0.37
LIM	0.74 (0.13–4.2)	−0.35	0.72
**LIMX**	**2.2 (1.1–4.5)**	**2.27**	**0.023**
SDEV	3.0 (0.82–13.0)	1.58	0.11
SIMX	1.4 (0.63–3.4)	0.84	0.40
**WB**	**0.20 (0.029–0.84)**	−**1.98**	**0.048**

Estimated parameters from the final selected logistic regression model for the probability of an animal possessing the ‘22’ genotype (p22 ∼ Age + *a2* + breed). Odds ratios are presented to two significant figures, along with 95% confidence intervals. Significant effects at the 95% level are highlighted in bold. The selected model shows no significant evidence of a lack of fit (p-value = 0.51). Predictive ability of the selected models was assessed using the receiver-operating-characteristic (ROC) curve, which has an area under the curve of 0.68. Breed effects are measured relative to the Holstein Breed (HOL) that is the most represented breed within the study population. For breed codes, see Table 1.

**Figure 2:**
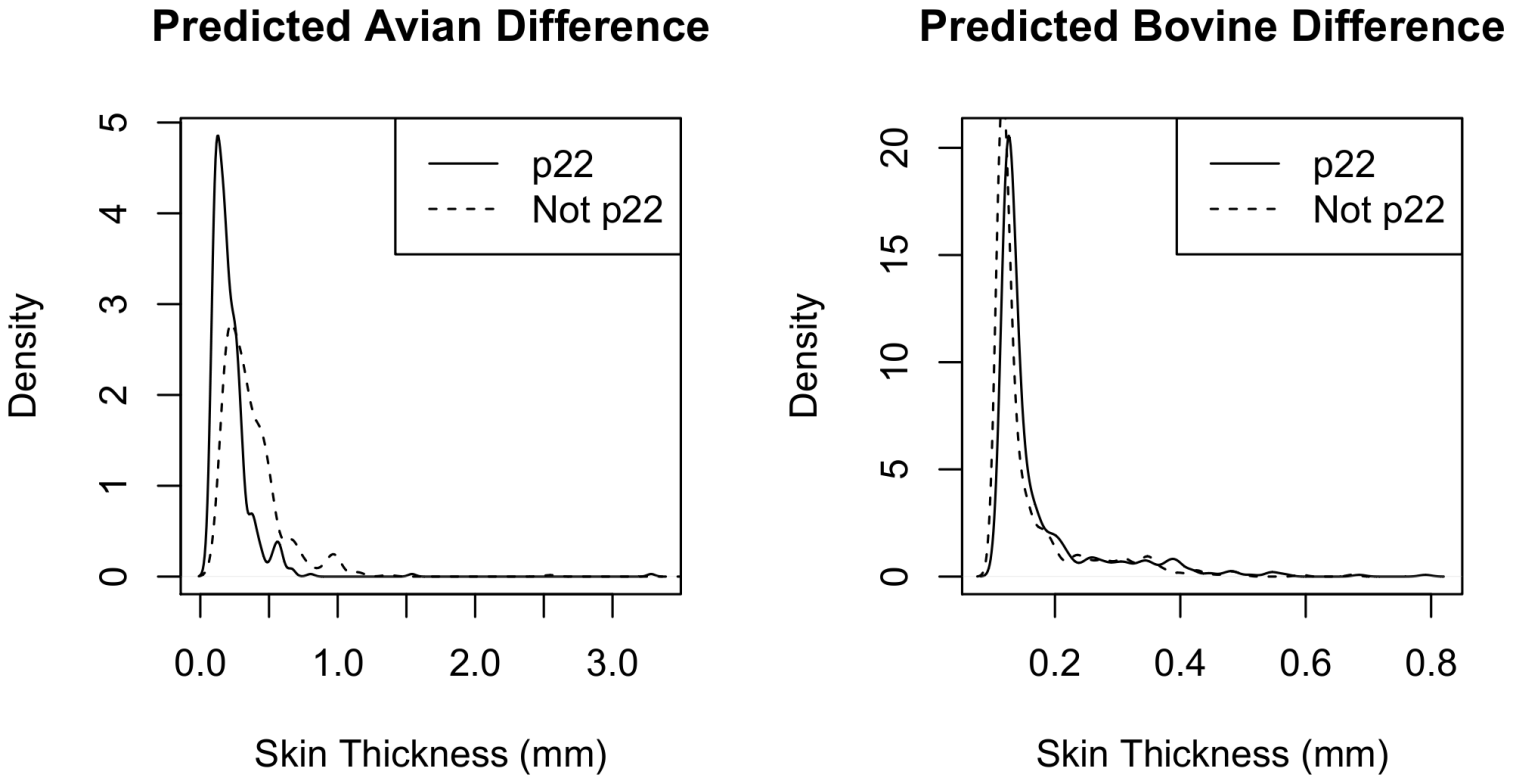
Predicted effect of ‘22’ genotype on the swelling induced by avian and bovine tuberculin challenges. Swelling size is taken as the difference between the initial measurement, taken immediately following injection, and the final measurement, taken after the prescribed 72 hour time delay that allows an immune response to occur (hereafter = ‘difference’). This controls for skin thickness differences between animals. The graphs show the predicted impact of the ‘22’ parameter (0 = not ‘22’, 1 = ‘22’) on the avian (da, left) and bovine (*db*, right) differences. Predicted values are calculated from the respective zero-inflated regression models *da* ∼ age + *a*1 + p22, *db* ∼ age + *b1* + p22 described within the main text (summarised in Tables 3 and 4). The distribution of predicted values with (solid line) and without (dashed line) the ‘22’ genotype are compared as smoothed density curves. No effect was found for the bovine differences but the model predicts a smaller avian difference (*da*) when among animals with the ‘22’ genotype.

To quantify the effect of ‘22’ status on the avian and bovine differences (*da*, *db*) we fitted two further regression models. Given the excess of zero counts for these variables, a standard Poisson regression model provides a poor fit to the data. We therefore use a zero-inflated Poisson model, which consists of a mixture distribution of a count model (Poisson) and a binomial (logit) model. Unfortunately, due to the limited number of samples within each breed (Table 1), breed cannot be included for this model using the current data set. We therefore use the first skin test measurement (*b1*, *a1*) as a proxy measure to correct for breed effects, regressing da ∼ Age + p22 + *a1* and *db* ∼ Age + p22 + *b1*. In line with the analysis for predictors of 22 status, we find a stronger association between the ‘22’ genotype and the avian measurements (Tables 3 and 4), with a predicted reduction in *da* with p22 (Figure 2). The zero inflated models provide a significantly better fit than the equivalent Poisson models (p = 3×10^−7^ for *da* and p = 3×10^−5^ for *db*) using the Vuong non-nested hypothesis test [32]. Due to the effective reduction in sample size resulting from the excess of zero measurements, the association for the difference measurements with p22 is only marginally significant at the 95% level for both models and we find no predicted effect of ‘22’ on the bovine difference (*db,* Figure 2).

**Table 3:** Prediction of difference in swelling size between initial and final measurements at the avian tuberculin injection site (*da*) by ‘22’ genotype.

Count model (Poisson)			
	Incident Risk Ratio	z value	Pr(>|z|)
**Age**	**1.0 (1.0–1.0)**	**−4.082**	**4.46e–05**
*a1*	1.0 (0.92–1.2)	0.834	0.4
**p22**	**0.7 (0.46–1.2)**	**−2.053**	**0.04**
**Zero-inflation model (Binomial)**			
	**Odds Ratio**	**z value**	**Pr(>|z|)**
**Age**	**1.0 (1.0–1.0)**	**−2.060**	**0.04**
***a1***	**0.84 (0.71–0.94)**	**−2.747**	**0.006**
p22	1.2 (0.53–2.9)	0.564	0.57

Incident risk and odds ratios for both components of a zero-inflated Poisson model fitted to the avian difference (*da* ∼ Age + *a1* + p22). Odds and incident risk ratios (from the Poisson count model and binomial zero inflation terms respectively) are presented to two significant figures, along with 95% confidence intervals calculated from 10000 parametric bootstraps. Significant effects at the 95% level are highlighted in bold. While the age co-efficient is highly significant with the Poisson portion of the model, the p22 effect is only marginally significant for *da*. The marginal significance of the p22 effect is further emphasised by the variability in the bootstrapped confidence interval, which constitutes a more conservative test.

**Table 4:** Prediction of difference in swelling size between initial and final measurements at the bovine tuberculin injection site (*db*) by ‘22’ genotype.

Count model (Poisson)			
Age	1.0 (1.0–1.0)	−0.254	0.7998
*b1*	1.0 (0.86–1.3)	0.560	0.5758
**p22**	**1.6 (0.82–4.0)**	**1.985**	**0.0472**
**Zero-inflation model (Binomial)**			
	**Odds Ratio**	**z value**	**Pr(>|z|)**
**Age**	**1.0 (1.0–1.0)**	**−2.191**	**0.0284**
*b1*	0.91 (0.76–1.1)	−1.280	0.2005
p22	1.44 (0.57–4.05)	0.841	0.4005

Incident risk and odds ratios for both components of a zero-inflated Poisson model fitted to the bovine difference (*db* ∼ Age + *b1* + p22). Odds and incident risk ratios (from the Poisson count model and binomial zero inflation terms respectively) are presented to two significant figures, along with 95% confidence intervals calculated from 10000 parametric bootstraps. Significant effects at the 95% level are highlighted in bold. While the age co-efficient is highly significant with the Poisson portion of the model, the p22 effect is only marginally significant for *da*. The marginal significance of the p22 effect is further emphasised by the variability in the bootstrapped confidence interval, which constitutes a more conservative test.

**Figure 3:**
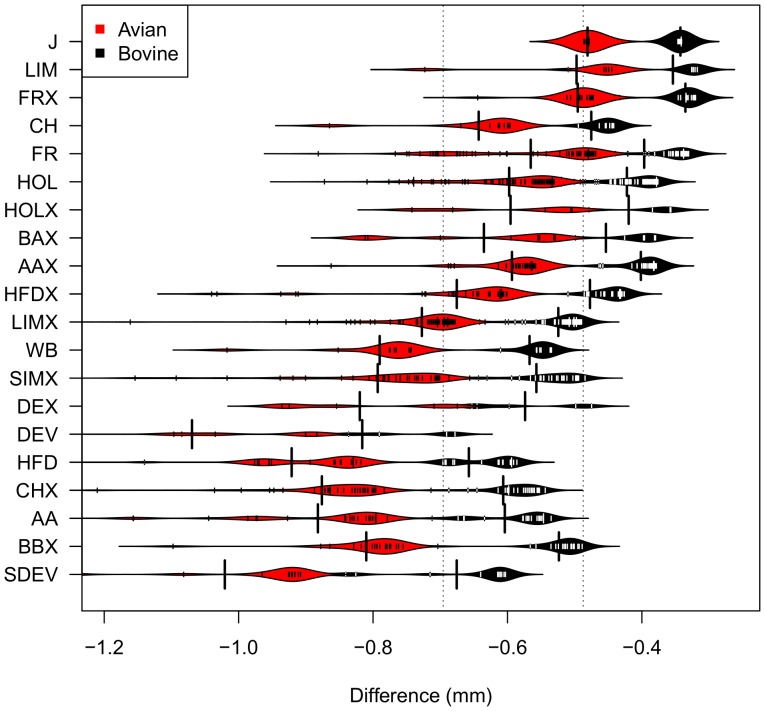
Predicted effect of ‘22’ genotype on second avian (*a2*, red) and second bovine (*b2*, black) measurements. The predicted impact of the ‘22’ parameter (0 = not ‘22’, 1 = ‘22’) on the second avian and second bovine swelling size measurements within our study population is summarised as a ‘beanplot’. The solid envelope represents the smoothed density kernel for the predicted values. Actual values are over-plotted as solid lines and the vertical dotted lines indicate the mean effect sizes across all breeds for the two measurements. Predicted values are calculated from the two Poisson regression models: *a2* ∼ p22 + breed + age; *b2* ∼ p22 + breed+age (Tables S2, S3).

To account for breed effects explicitly, we finally fit two generalised linear models (glms) with a Poisson error structure with *b2* and *a2* as the response variables and breed, age and ‘22’ status as predictors. Both models demonstrate a significant effect of ‘22’ status on the second bovine (p = 0.042) and avian measurements (p = 0.0039) after accounting for breed and age effects (Tables S2, S3). We use the fitted models to predict the impact of ‘22’ status on the bovine and avian bump sizes (Figure 3). Overall, ‘22’ animals are predicted to have a smaller average bump size, with a greater reduction for the avian measurement than for the bovine measurement across all breeds. The predicted average effect sizes for bovine and avian swelling sizes are reductions of 0.4 mm and 0.7 mm respectively. These are not large compared with either the precision of skin measurements (1 mm) or the threshold required to condemn an animal as a reactor (4 mm), but may be sufficient to affect the test outcome of the many cattle with borderline swelling sizes.

## Discussion

In this paper, we explore evidence for a genetic effect that results in reduced responsiveness to the standard SICCT test, the test at the heart of many bovine tuberculosis control programmes internationally. We find a strong association between breed and test outcomes, with smaller reactions in the common dairy breeds Jersey, Friesian and Holstein, and larger reactions in various beef breeds and their crossbreds. Furthermore, building on previous work that demonstrated an over-representation of marker INRA111 ‘22’ genotypes among non-reactors, we present evidence that the putative protective ‘22’ genotype acts, at least in part, by producing smaller swellings at the injection site in animals then classified as non-reactors and a reduced difference between the reaction to bovine and avian tuberculin challenges at first test. Possible implications are: (a) that some cattle may have a decreased propensity to progress to infectious disease once exposed; (b) that they escape detection in the early stages of infection; or both may be occurring. These findings present a new angle on the epidemiology of bovine TB in Great Britain and further work is warranted to understand the important relationship between reactivity and infectiousness and the resulting impact for any ‘test-and-slaughter’ policy.

In theory, rapid early detection and removal of diseased individuals from a population should provide an effective method of disease eradication. However, in Great Britain there is evidence of a high level of within-herd persistence with approximately 38% of herds certified as officially TB free at the end of an outbreak going on to experience a recurrent incident within 24 months [2]. Persistence is likely to be facilitated by failure to detect infected cattle sufficiently early to prevent the infection of others. This is a well-known problem, with the standard SICCT test sensitivity estimated to only be able to detect around 40–80% of infected animals [4], [33], [34]. Furthermore, the current herd-based testing policy may leave 70–80% of cattle not tested during their lifetimes [33], though it should be noted that many of these untested animals are non-breeding animals in low risk areas of the country. Recent within-herd modelling suggests that up to 50% of recurrent breakdowns can be attributed to infection missed by testing, with up to 21% of herds likely to be harbouring infection after clearing movement restrictions [4]. However, there is also evidence of a considerable rate of reintroduction of infection into herds through cattle movements [35] or from wildlife reservoirs. Our study identifies a third factor in preventing early detection, namely that a subset of cattle may be genetically less responsive to the standard SICCT test.

Our results help to document some of the components of variability associated with the SICCT test, including extensive variation in average swelling size between breeds and among animals of different ages. Swelling size also seems impacted by interactions between breed and age. Previous risk factor analyses have demonstrated an increased risk of reacting to the SICCT test associated with dairy breeds and older animals [36], [37]. Breeds differ appreciably in muscle definition, fat composition, length and structure of hair (though this is clipped prior to testing) and other traits, including bTB pathology [38], and it is therefore not surprising that across a range of contrasting hides swelling size measurements to the nearest millimetre vary significantly. Variation between breeds will also result from metabolic stress, differences in movement patterns and life histories. The current SICCT test protocol explicitly adjusts for this variation by defining diagnostic status in terms of the difference relative to an initial skin thickness measurement (*a1*, *b1*). Variation in reaction sizes between breeds could be used to justify breed-specific thresholds for diagnosis.

It is plausible that a test-and-slaughter policy could select for a reduced cell-mediated immune response (CMIR). Studies in natural populations have revealed a relationship between inbreeding coefficient and CMIR [39]. More directly with respect to bovines, studies in Holstein cattle show that CMIR varies between individuals [40] and has an estimated heritability of 0.19 [41]. Together, these observations uncover the potential for selection to have favoured individuals with a reduced or altered CMIR that in turn would enable some infected cattle to pass the test, at least in the early stages of infection. Just how large an effect this would create and over what timescales is unclear. Test-and-slaughter has been in operation since the 1950s, over which time substantial changes in the breed composition of the national herd have occurred. The effect of test-and-slaughter on genotype frequencies would require detailed mathematical modelling for which many important parameters are unknown, including: the relationship between infectiousness and test status; the degree of protection afforded by being ‘22’; and specific breeding practices in GB. We particularly lack breed-specific life history curves that would allow estimation of the impact of delayed detection on probability of calving and any possible downstream impact of reduced CMIR on infectivity. Moreover, the differential impact on swelling generated by the avian and bovine challenges suggests a complicated relationship with the test outcome, with the generally smaller swellings reducing the chance of failing around the borderline but the relatively greater reduction in avian swelling causing more definitive failure once the bovine swelling becomes large enough.

At present, the impact of being a ‘22’ genotype appears rather modest. However, there are several reasons why this is likely to be an under-estimate. Most importantly, we are using a binary genotype classification, ‘22’ versus not ‘22’, at a microsatellite marker INRA111. In reality, this microsatellite is unlikely to be the functional variant but instead merely reflects the genotype at a neighbouring gene through linkage disequilibrium. Indeed, there is an excellent candidate gene, vaccinia related kinase 2 (VRK2), that is both the nearest gene to the microsatellite, lying 238.5 Kb on the 3′ side, and is a regulator of interleukin 1B [20], an important mediator of the inflammatory response [42]. Any correlation between genotype at a marker and genotype at an adjacent gene is invariably imperfect [43], and in most cases will be relatively weak, even when recombination rates between the two are negligible. This is because, unless the mutation causing the functional gene variant and a unique microsatellite mutation occur simultaneously, any given microsatellite allele will only ever ‘mark’ a subset of chromosomes carrying the functional variant. Consequently, the effect size we document is likely to increase appreciably were we able to genotype the functional variant in the gene itself.

A second reason why our observed effect size is unlikely to be large is sample size and composition. Our sampling strategy has been based on opportunistic collection of abattoir samples, providing logistic convenience and access to many breeds. Unfortunately, this leads to sample sizes for some breeds being rather small, an issue that is exacerbated by the fact that only approximately one in three of our samples has full test results in VeBus (nationally, this figure is more like one in ten, but is higher in high risk parts of the country like where our work was conducted). The upside of our strategy is that we are able to compare many different breeds and hence to detect patterns that are much more obvious in some breeds more than others. The INRA111 ‘22’ genotype effect is an interesting case in question because a difference in frequency between reactors and non-reactors is seen most strikingly in certain beef breeds but is reduced or even absent in the main dairy breeds that would be the logical choice for a more directed study. This does not necessarily mean that ‘22’ dairy cattle show no effect; it may be more that in dairy cattle a reduced swelling size merely delays the inevitable, with ‘22’ frequencies among reactors and non-reactors ending up very similar. In contrast, appreciable numbers of infected ‘22’ beef cattle may be slaughtered as non-reactors before they reach the stage when they fail the test.

Finally, the impact of ‘22’ genotype on swelling size appears to be rather complicated. Although the second swelling measurement is on average reduced across both injection sites and all breeds tested, the impact seems greater on avian than on the bovine tuberculin challenge, reflected both in the individual second measurements and in the fact that the reduction in measurement difference is significant only for the avian challenge. Quite why this should be remains unclear. The implications for the SICCT test are also not simple. Close to the pass-fail threshold ‘22’ animals are likely to benefit from the small reduction in *b2* swelling size. However, as soon as the *b2* threshold is exceeded, ‘22’ animals may be more likely to fail by dint of their reduced *a2* swelling size.

In conclusion, we provide the first evidence for genetic factors influencing the outcome of the standard SICCT test, a key tool in management strategies aimed at preventing the spread of bovine tuberculosis. Our results support previous work that identified an association between bTB and marker INRA111 and have direct implications for the effectiveness of the current test-and-slaughter policy, with the potential to prolong outbreaks on individual farms. Estimating the epidemiological impact of this effect will require detailed epidemiological modelling, though there are good reasons for believing the effect size we report is an under-estimate. Understanding the extent to which the INRA111 ‘22’ genotype influences the SICCT test result could help both to increase the statistical power of other on-going studies looking for genetic factors that affect susceptibility and to pave the way to the development of a more reliable test.

## Supporting Information

Table S1 **Average skin thickness recorded in VeBus for breeds within study population.** For breed codes see Table 1.
(DOCX)

Table S2 **Prediction of avian skin thickness measurement (a2) by ‘22’ genotype.** Summary of a Poisson error structure regression model exploring the association between having the ‘22’ genotype and size of the second avian skin thickness measurement (a2). Coefficients are reported to 2 significant figures, with 95% confidence intervals. Significant associations at the 95% level are highlighted in bold. Breed effects are measured relative to the Holstein Breed (HOL) that is the most represented breed within the study population.
(DOCX)

Table S3 **Prediction of bovine skin thickness measurement (b2) by ‘22’ genotype.** Summary of a Poisson error structure regression model exploring the association between having the ‘22’ genotype and size of the second bovine skin thickness measurement (b2). Coefficients are reported to 2 significant figures, with 95% confidence intervals. Significant associations at the 95% level are highlighted in bold. Breed effects are measured relative to the Holstein Breed (HOL) that is the most represented breed within the study population.
(DOCX)

Table S4 **Data set used for fitting models.** Columns are: # = sample number; Age = age at slaughter in days; Breed, given as the breed code, see Table 1; a1, a2, b1 and b2 are the four swelling size measurements given as first (1) and second (2) for the avian (a) and bovine (b) injection sites; da and db are the swelling size differences, given as the second minus the first swelling size measurements at the avian (da) and bovine (db) injection sites; status = R for reactor and NR for non-reactor; p22 = genotype at microsatellite INRA111 with TRUE = ‘22’ and FALSE = not ‘22’.
(DOCX)
