# Supplementary material for: Genetic Predisposition to Pass the Standard SICCT Test for Bovine Tuberculosis in British Cattle
Source: PLoS One. 2013 Mar 6;8(3):e58245. doi: 10.1371/journal.pone.0058245 (PMC3605902; doi:10.1371/journal.pone.0058245)
Supplement: Table S1 — Average skin thickness recorded in VeBus for breeds within study population. For breed codes see Table 1. (DOCX) [file pone.0058245.s001.docx]

**Table S1: Average skin thickness recorded in VeBus for breeds within study population.**

| Breed | Records | Average Skin Thickness (mm) |
| --- | --- | --- |
| WB | 35842 | 8.05 (0.01) |
| DEV | 14234 | 7.76 (0.02) |
| HFD | 36786 | 7.57 (0.01) |
| CH | 533346 | 7.53 (0.01) |
| AA | 52132 | 7.51 (0.01) |
| DEX | 12115 | 7.44 (0.02) |
| SDEV | 30170 | 7.25 (0.01) |
| LIM | 130389 | 7.14 (0.007) |
| SIMX | 203236 | 7.11 (0.005) |
| CHX | 337358 | 6.96 (0.003) |
| BAX | 77861 | 6.91 (0.008) |
| LIMX | 662508 | 6.82 (0.002) |
| HFDX | 159484 | 6.72 (0.005) |
| AAX | 171939 | 6.65 (0.005) |
| BBX | 209025 | 6.41 (0.004) |
| FR | 303468 | 6.04 (0.003) |
| HOLX | 66092 | 6.03 (0.07) |
| HOL | 989646 | 6.02 (0.002) |
| FRX | 37962 | 5.72 (0.009) |
| J | 32254 | 5.59 (0.01) |
