# Supplementary material for: Genetic Predisposition to Pass the Standard SICCT Test for Bovine Tuberculosis in British Cattle
Source: PLoS One. 2013 Mar 6;8(3):e58245. doi: 10.1371/journal.pone.0058245 (PMC3605902; doi:10.1371/journal.pone.0058245)
Supplement: Table S2 — Prediction of avian skin thickness measurement (a2) by ‘22’ genotype. Summary of a Poisson error structure regression model exploring the association between having the ‘22’ genotype and size of the second avian skin thickness measurement (a2). Coefficients are reported to 2 significant figures, with 95% confidence intervals. Significant associations at the 95% level are highlighted in bold. Breed effects are measured relative to the Holstein Breed (HOL) that is the most represented breed within the study population. (DOCX) [file pone.0058245.s002.docx]

**Table S2: Prediction of avian skin thickness measurement (a2) by ‘22’ genotype.**

|  | **Co-efficient (95% CI)** | **z value** | **Pr(>\|z\|)** |
| --- | --- | --- | --- |
| (Intercept) | 5.4 (4.9-5.9) | 34.28 | < 2e-16 |
| **Age** | **1.0 (1.0-1.0)** | **8.14** | **4.1e-16** |
| **p22** | **0.9 (0.84-0.97)** | **-2.89** | **0.004** |
| **AA** | **1.5 (1.3-1.8)** | **4.84** | **1.3e-06** |
| AAX | 1.1 (0.90-1.2) | 0.64 | 0.52 |
| BAX | 1.0 (0.74-1.3) | -0.01 | 0.99 |
| **BBX** | **1.4 (1.2-1.7)** | **3.93** | **8.4e-05** |
| CH | 1.1 (0.84-1.45) | 0.79 | 0.43 |
| **CHX** | **1.5 (1.3-1.72)** | **5.62** | **1.9e-08** |
| **DEV** | **1.6 (1.2-2.1)** | **3.23** | **0.001** |
| DEX | 1.2 (0.85-1.6) | 1.09 | 0.28 |
| FR | 0.88 (0.77-1.0) | -1.76 | 0.08 |
| FRX | 0.89 (0.70-1.1) | -1.05 | 0.30 |
| **HFD** | **1.5 (1.3-1.8)** | **4.89** | **1.0e-06** |
| HFDX | 1.1 (0.97-1.3) | 1.64 | 0.10 |
| HOLX | 0.95 (0.6-1.4) | -0.28 | 0.78 |
| J | 0.90 (0.66-1.2) | -0.71 | 0.48 |
| LIM | 0.84 (0.56-1.2) | -0.93 | 0.30 |
| **LIMX** | **1.3 (1.1-1.4)** | **3.86** | **0.0001** |
| **SDEV** | **1.7 (1.4-2.0)** | **5.28** | **1.3e-07** |
| **SIMX** | **1.3 (1.1-1.5)** | **3.65** | **0.0003** |
| **WB** | **1.4 (1.1-1.7)** | **2.98** | **0.003** |
