# Supplementary material for: Genetic Predisposition to Pass the Standard SICCT Test for Bovine Tuberculosis in British Cattle
Source: PLoS One. 2013 Mar 6;8(3):e58245. doi: 10.1371/journal.pone.0058245 (PMC3605902; doi:10.1371/journal.pone.0058245)
Supplement: Table S3 — Prediction of bovine skin thickness measurement (b2) by ‘22’ genotype. Summary of a Poisson error structure regression model exploring the association between having the ‘22’ genotype and size of the second bovine skin thickness measurement (b2). Coefficients are reported to 2 significant figures, with 95% confidence intervals. Significant associations at the 95% level are highlighted in bold. Breed effects are measured relative to the Holstein Breed (HOL) that is the most represented breed within the study population. (DOCX) [file pone.0058245.s003.docx]

**Table S3: Prediction of bovine skin thickness measurement (b2) by ‘22’ genotype.**

|  | **Co-efficient (95% CI)** | **z value** | **Pr(>\|z\|)** |
| --- | --- | --- | --- |
| (Intercept) | 5.3 (4.8-5.8) | 33. 8 | < 2e-16 |
| **Age** | **1.0 (1.0-1.0)** | **7.87** | **3.5e-15** |
| **p22** | **0.93 (0.87-1.0)** | **-2.04** | **0.04** |
| **AA** | **1.5 (1.2-1.7)** | **4.41** | **1.0e-05** |
| AAX | 1.0 (0.85-1.2) | 0.09 | 0.93 |
| BAX | 1.0 (0.75-1.30) | 0.07 | 0.94 |
| **BBX** | **1.3 (1.1-1.6)** | **2.81** | **0.005** |
| CH | 1.2 (0.88-1.5) | 1.13 | 0.26 |
| **CHX** | **1.5 (1.3-1.7)** | **5.27** | **1.3e-07** |
| **DEV** | **1.7 (1.3-2.27)** | **3.91** | **9.4e-05** |
| DEX | 1.2 (0.85-1.6) | 1.04 | 0.3 |
| FR | 0.88 (0.77-1.0) | -1.84 | 0.07 |
| FRX | 0.85 (0.67-1.1) | -1.42 | 0.16 |
| **HFD** | **1.6 (1.3-1.8)** | **5.00** | **5.8e-07** |
| HFDX | 1.1 (0.97-1.3) | 1.63 | 0.1 |
| HOLX | 0.94 (0.63-1.3) | -0.29 | 0.77 |
| J | 0.90 (0.66-1.2) | -0.67 | 0.50 |
| LIM | 0.84 (0.57-1.2) | -0.89 | 0.37 |
| **LIMX** | **1.3 (1.1-1.5)** | **4.17** | **3.1e-05** |
| **SDEV** | **1.6 (1.3-1.9)** | **4.49** | **7.0e-06** |
| **SIMX** | **1.3 (1.1-1.5)** | **3.56** | **0.0004** |
| **WB** | **1.4 (1.1-1.7)** | **3.10** | **0.002** |
