# Supplementary material for: Genetic Predisposition to Pass the Standard SICCT Test for Bovine Tuberculosis in British Cattle
Source: PLoS One. 2013 Mar 6;8(3):e58245. doi: 10.1371/journal.pone.0058245 (PMC3605902; doi:10.1371/journal.pone.0058245)
Supplement: Table S4 — Data set used for fitting models. Columns are: # = sample number; Age = age at slaughter in days; Breed, given as the breed code, see Table 1; a1, a2, b1 and b2 are the four swelling size measurements given as first (1) and second (2) for the avian (a) and bovine (b) injection sites; da and db are the swelling size differences, given as the second minus the first swelling size measurements at the avian (da) and bovine (db) injection sites; status = R for reactor and NR for non-reactor; p22 = genotype at microsatellite INRA111 with TRUE = ‘22’ and FALSE = not ‘22’. (DOCX) [file pone.0058245.s004.docx]

**Table S4: Data set used for fitting models.**

| **#** | **Age** | **Breed** | **a1** | **b1** | **a2** | **b2** | **da** | **db** | **status** | **p22** |
| --- | --- | --- | --- | --- | --- | --- | --- | --- | --- | --- |
| 1 | 797 | HOL | 6 | 6 | 6 | 6 | 0 | 0 | R | TRUE |
| 2 | 280 | HFDX | 5 | 5 | 5 | 5 | 0 | 0 | NR | FALSE |
| 3 | 302 | HOL | 5 | 5 | 6 | 6 | 1 | 1 | R | FALSE |
| 4 | 293 | AAX | 8 | 8 | 8 | 8 | 0 | 0 | R | TRUE |
| 5 | 1833 | LIMX | 8 | 8 | 8 | 8 | 0 | 0 | R | TRUE |
| 6 | 1170 | HFD | 18 | 18 | 18 | 18 | 0 | 0 | NR | FALSE |
| 7 | 1030 | HFD | 14 | 14 | 14 | 14 | 0 | 0 | NR | FALSE |
| 8 | 1056 | HFD | 12 | 12 | 12 | 12 | 0 | 0 | NR | FALSE |
| 9 | 1154 | HFD | 8 | 8 | 8 | 8 | 0 | 0 | NR | FALSE |
| 10 | 1200 | HFD | 8 | 8 | 12 | 14 | 4 | 6 | NR | TRUE |
| 11 | 1054 | HFD | 8 | 8 | 12 | 8 | 4 | 0 | NR | TRUE |
| 12 | 1918 | FR | 8 | 8 | 10 | 8 | 2 | 0 | R | FALSE |
| 13 | 753 | DEX | 11 | 10 | 11 | 10 | 0 | 0 | R | TRUE |
| 14 | 2752 | HFDX | 8 | 8 | 8 | 8 | 0 | 0 | R | TRUE |
| 15 | 2727 | HFDX | 6 | 6 | 6 | 6 | 0 | 0 | R | TRUE |
| 16 | 812 | CH | 10 | 10 | 10 | 10 | 0 | 0 | R | TRUE |
| 17 | 1156 | HOL | 6 | 8 | 6 | 8 | 0 | 0 | NR | TRUE |
| 18 | 899 | HOL | 6 | 5 | 7 | 7 | 1 | 2 | R | FALSE |
| 19 | 2915 | SIMX | 6 | 6 | 6 | 6 | 0 | 0 | R | TRUE |
| 20 | 3271 | SIMX | 6 | 6 | 6 | 6 | 0 | 0 | R | TRUE |
| 21 | 766 | LIMX | 13 | 13 | 13 | 13 | 0 | 0 | NR | TRUE |
| 22 | 764 | LIMX | 13 | 13 | 13 | 13 | 0 | 0 | NR | FALSE |
| 23 | 2186 | HOL | 5 | 5 | 5 | 5 | 0 | 0 | NR | FALSE |
| 24 | 2197 | HOL | 7 | 7 | 9 | 7 | 2 | 0 | NR | FALSE |
| 25 | 828 | HOL | 8 | 8 | 8 | 8 | 0 | 0 | NR | FALSE |
| 26 | 341 | HFD | 8 | 8 | 8 | 8 | 0 | 0 | R | FALSE |
| 27 | 100 | HFD | 6 | 7 | 6 | 7 | 0 | 0 | R | FALSE |
| 28 | 125 | CHX | 6 | 6 | 6 | 6 | 0 | 0 | R | TRUE |
| 29 | 202 | SDEV | 11 | 11 | 11 | 11 | 0 | 0 | NR | FALSE |
| 30 | 176 | SDEV | 12 | 12 | 20 | 13 | 8 | 1 | NR | TRUE |
| 31 | 127 | LIMX | 4 | 4 | 4 | 4 | 0 | 0 | NR | FALSE |
| 32 | 317 | LIMX | 6 | 6 | 6 | 6 | 0 | 0 | NR | TRUE |
| 33 | 86 | HFDX | 4 | 4 | 4 | 4 | 0 | 0 | NR | TRUE |
| 34 | 115 | HFDX | 4 | 4 | 4 | 4 | 0 | 0 | NR | TRUE |
| 35 | 176 | LIMX | 6 | 6 | 6 | 6 | 0 | 0 | NR | TRUE |
| 36 | 230 | FR | 4 | 4 | 4 | 4 | 0 | 0 | R | FALSE |
| 37 | 480 | FR | 4 | 4 | 4 | 4 | 0 | 0 | R | TRUE |
| 38 | 141 | FR | 3 | 3 | 3 | 3 | 0 | 0 | R | TRUE |
| 39 | 157 | FR | 3 | 3 | 3 | 3 | 0 | 0 | R | FALSE |
| 40 | 154 | FR | 3 | 3 | 3 | 3 | 0 | 0 | R | TRUE |
| 41 | 207 | FR | 4 | 4 | 4 | 4 | 0 | 0 | R | TRUE |
| 42 | 229 | FR | 4 | 4 | 4 | 4 | 0 | 0 | R | FALSE |
| 43 | 196 | FR | 4 | 4 | 4 | 4 | 0 | 0 | R | TRUE |
| 44 | 167 | FR | 3 | 3 | 3 | 3 | 0 | 0 | R | FALSE |
| 45 | 8 | FR | 3 | 3 | 3 | 3 | 0 | 0 | R | TRUE |
| 46 | 1211 | HFDX | 8 | 8 | 8 | 8 | 0 | 0 | R | FALSE |
| 47 | 218 | FR | 4 | 4 | 4 | 4 | 0 | 0 | R | FALSE |
| 48 | 47 | FR | 3 | 3 | 3 | 3 | 0 | 0 | R | FALSE |
| 49 | 127 | FR | 3 | 3 | 3 | 3 | 0 | 0 | R | TRUE |
| 50 | 6 | FR | 3 | 3 | 3 | 3 | 0 | 0 | R | TRUE |
| 51 | 485 | FR | 4 | 4 | 4 | 4 | 0 | 0 | R | FALSE |
| 52 | 390 | FR | 4 | 4 | 4 | 4 | 0 | 0 | R | FALSE |
| 53 | 342 | FR | 4 | 4 | 4 | 4 | 0 | 0 | R | FALSE |
| 54 | 164 | FR | 3 | 3 | 3 | 3 | 0 | 0 | R | TRUE |
| 55 | 355 | FR | 4 | 4 | 4 | 4 | 0 | 0 | R | FALSE |
| 56 | 356 | HOL | 5 | 5 | 5 | 5 | 0 | 0 | R | TRUE |
| 57 | 132 | J | 5 | 5 | 5 | 5 | 0 | 0 | R | TRUE |
| 58 | 54 | J | 4 | 4 | 4 | 4 | 0 | 0 | R | FALSE |
| 59 | 48 | J | 4 | 4 | 4 | 4 | 0 | 0 | R | FALSE |
| 60 | 54 | J | 5 | 5 | 5 | 5 | 0 | 0 | R | FALSE |
| 61 | 69 | J | 5 | 5 | 5 | 5 | 0 | 0 | R | FALSE |
| 62 | 59 | J | 5 | 5 | 5 | 5 | 0 | 0 | R | TRUE |
| 63 | 151 | J | 4 | 4 | 4 | 4 | 0 | 0 | R | TRUE |
| 64 | 420 | SIMX | 10 | 10 | 10 | 10 | 0 | 0 | NR | FALSE |
| 65 | 215 | CHX | 4 | 4 | 4 | 4 | 0 | 0 | NR | TRUE |
| 66 | 68 | CHX | 5 | 5 | 5 | 5 | 0 | 0 | NR | TRUE |
| 67 | 220 | CHX | 7 | 7 | 7 | 7 | 0 | 0 | R | TRUE |
| 68 | 924 | SIMX | 11 | 11 | 11 | 11 | 0 | 0 | R | TRUE |
| 69 | 175 | LIMX | 7 | 7 | 7 | 7 | 0 | 0 | NR | TRUE |
| 70 | 173 | LIMX | 9 | 9 | 9 | 9 | 0 | 0 | NR | TRUE |
| 71 | 151 | LIMX | 7 | 8 | 7 | 8 | 0 | 0 | NR | TRUE |
| 72 | 5 | HFDX | 7 | 7 | 7 | 7 | 0 | 0 | NR | FALSE |
| 73 | 270 | HFDX | 5 | 5 | 5 | 5 | 0 | 0 | NR | TRUE |
| 74 | 243 | DEV | 9 | 12 | 9 | 12 | 0 | 0 | NR | TRUE |
| 75 | 3265 | HOL | 7 | 7 | 7 | 7 | 0 | 0 | R | TRUE |
| 76 | 104 | HFDX | 4 | 4 | 4 | 4 | 0 | 0 | NR | FALSE |
| 77 | 63 | HFDX | 4 | 4 | 4 | 4 | 0 | 0 | NR | FALSE |
| 78 | 1921 | HOL | 4 | 4 | 4 | 4 | 0 | 0 | R | TRUE |
| 79 | 324 | BBX | 6 | 6 | 6 | 6 | 0 | 0 | NR | FALSE |
| 80 | 96 | AA | 5 | 5 | 5 | 5 | 0 | 0 | NR | TRUE |
| 81 | 183 | LIMX | 7 | 7 | 7 | 7 | 0 | 0 | NR | TRUE |
| 82 | 583 | FR | 6 | 6 | 6 | 6 | 0 | 0 | R | FALSE |
| 83 | 1650 | SIMX | 7 | 7 | 7 | 7 | 0 | 0 | R | TRUE |
| 84 | 130 | SIMX | 6 | 8 | 6 | 8 | 0 | 0 | R | TRUE |
| 85 | 727 | LIMX | 7 | 7 | 7 | 7 | 0 | 0 | R | TRUE |
| 86 | 288 | BAX | 6 | 6 | 6 | 6 | 0 | 0 | NR | TRUE |
| 87 | 39 | HOL | 5 | 5 | 5 | 5 | 0 | 0 | R | TRUE |
| 88 | 176 | LIMX | 6 | 6 | 6 | 6 | 0 | 0 | R | TRUE |
| 89 | 156 | BBX | 4 | 4 | 19 | 5 | 15 | 1 | R | FALSE |
| 90 | 169 | BBX | 5 | 5 | 5 | 5 | 0 | 0 | R | FALSE |
| 91 | 61 | J | 6 | 6 | 6 | 6 | 0 | 0 | R | TRUE |
| 92 | 289 | HFDX | 5 | 6 | 5 | 6 | 0 | 0 | NR | TRUE |
| 93 | 74 | LIMX | 4 | 4 | 4 | 4 | 0 | 0 | NR | FALSE |
| 94 | 1052 | HOL | 6 | 6 | 6 | 6 | 0 | 0 | NR | TRUE |
| 95 | 279 | HOL | 4 | 4 | 4 | 4 | 0 | 0 | NR | TRUE |
| 96 | 829 | HOL | 6 | 6 | 6 | 6 | 0 | 0 | NR | TRUE |
| 97 | 801 | HOL | 8 | 8 | 8 | 8 | 0 | 0 | NR | TRUE |
| 98 | 1057 | HOL | 6 | 6 | 6 | 6 | 0 | 0 | NR | TRUE |
| 99 | 151 | HOL | 4 | 4 | 4 | 4 | 0 | 0 | R | TRUE |
| 100 | 808 | HOL | 6 | 6 | 6 | 6 | 0 | 0 | R | TRUE |
| 101 | 1428 | HOL | 8 | 8 | 8 | 8 | 0 | 0 | R | TRUE |
| 102 | 115 | SDEV | 6 | 6 | 6 | 6 | 0 | 0 | R | FALSE |
| 103 | 201 | SDEV | 6 | 6 | 6 | 6 | 0 | 0 | R | TRUE |
| 104 | 248 | SIMX | 12 | 13 | 12 | 13 | 0 | 0 | R | TRUE |
| 105 | 14 | SIMX | 7 | 7 | 7 | 7 | 0 | 0 | R | TRUE |
| 106 | 75 | HOL | 4 | 4 | 4 | 4 | 0 | 0 | R | TRUE |
| 107 | 1369 | AAX | 8 | 7 | 8 | 7 | 0 | 0 | R | TRUE |
| 108 | 1753 | CHX | 9 | 9 | 9 | 9 | 0 | 0 | R | FALSE |
| 109 | -73 | CHX | 9 | 9 | 9 | 9 | 0 | 0 | R | FALSE |
| 110 | 414 | CHX | 9 | 9 | 9 | 9 | 0 | 0 | R | FALSE |
| 111 | 283 | CHX | 8 | 8 | 8 | 8 | 0 | 0 | R | FALSE |
| 112 | 4 | BAX | 3 | 3 | 3 | 3 | 0 | 0 | R | FALSE |
| 113 | 535 | HOL | 6 | 6 | 6 | 6 | 0 | 0 | R | TRUE |
| 114 | 65 | SIMX | 7 | 7 | 7 | 7 | 0 | 0 | R | FALSE |
| 115 | 57 | SIMX | 4 | 4 | 4 | 4 | 0 | 0 | R | FALSE |
| 116 | 27 | SIMX | 3 | 3 | 3 | 3 | 0 | 0 | R | TRUE |
| 117 | 33 | BAX | 2 | 2 | 2 | 2 | 0 | 0 | NR | FALSE |
| 118 | 65 | AAX | 4 | 4 | 4 | 4 | 0 | 0 | NR | TRUE |
| 119 | 39 | WB | 6 | 6 | 6 | 6 | 0 | 0 | NR | TRUE |
| 120 | 51 | WB | 6 | 6 | 6 | 6 | 0 | 0 | NR | TRUE |
| 121 | 49 | WB | 3 | 3 | 3 | 3 | 0 | 0 | NR | FALSE |
| 122 | 51 | HOL | 4 | 4 | 4 | 4 | 0 | 0 | R | FALSE |
| 123 | 15 | HOL | 4 | 4 | 4 | 4 | 0 | 0 | R | TRUE |
| 124 | 48 | HOL | 4 | 4 | 4 | 4 | 0 | 0 | R | FALSE |
| 125 | 37 | HOLX | 4 | 4 | 4 | 4 | 0 | 0 | R | TRUE |
| 126 | 20 | HOL | 4 | 4 | 4 | 4 | 0 | 0 | R | TRUE |
| 127 | 33 | HOL | 4 | 4 | 4 | 4 | 0 | 0 | R | TRUE |
| 128 | 188 | HOL | 6 | 6 | 6 | 6 | 0 | 0 | R | TRUE |
| 129 | 533 | HFDX | 6 | 6 | 6 | 6 | 0 | 0 | R | FALSE |
| 130 | 789 | HFDX | 12 | 12 | 22 | 19 | 10 | 7 | NR | FALSE |
| 131 | 60 | HFDX | 4 | 4 | 4 | 4 | 0 | 0 | NR | FALSE |
| 132 | 45 | HFD | 4 | 4 | 4 | 4 | 0 | 0 | NR | TRUE |
| 133 | 1969 | DEX | 7 | 7 | 7 | 7 | 0 | 0 | R | FALSE |
| 134 | 465 | HFDX | 7 | 7 | 7 | 7 | 0 | 0 | NR | FALSE |
| 135 | 471 | HFDX | 7 | 7 | 7 | 7 | 0 | 0 | NR | FALSE |
| 136 | 386 | FR | 7 | 7 | 7 | 7 | 0 | 0 | R | TRUE |
| 137 | 1762 | HOL | 7 | 7 | 7 | 7 | 0 | 0 | R | TRUE |
| 138 | 1918 | SIMX | 11 | 11 | 11 | 11 | 0 | 0 | R | FALSE |
| 139 | 270 | LIMX | 8 | 8 | 8 | 8 | 0 | 0 | R | FALSE |
| 140 | 80 | HFDX | 5 | 5 | 5 | 5 | 0 | 0 | NR | TRUE |
| 141 | 65 | HFDX | 6 | 6 | 6 | 6 | 0 | 0 | NR | FALSE |
| 142 | 111 | AAX | 4 | 4 | 4 | 4 | 0 | 0 | NR | TRUE |
| 143 | 86 | AAX | 4 | 4 | 4 | 4 | 0 | 0 | NR | TRUE |
| 144 | 76 | AAX | 4 | 4 | 4 | 4 | 0 | 0 | NR | TRUE |
| 145 | 66 | HFDX | 4 | 4 | 4 | 4 | 0 | 0 | NR | FALSE |
| 146 | 57 | LIMX | 5 | 5 | 5 | 5 | 0 | 0 | NR | TRUE |
| 147 | 50 | LIMX | 4 | 4 | 4 | 4 | 0 | 0 | R | FALSE |
| 148 | 1239 | FR | 8 | 8 | 8 | 8 | 0 | 0 | R | TRUE |
| 149 | 149 | HOL | 6 | 6 | 6 | 6 | 0 | 0 | R | TRUE |
| 150 | 376 | CH | 10 | 10 | 10 | 10 | 0 | 0 | R | FALSE |
| 151 | 223 | CH | 6 | 6 | 6 | 6 | 0 | 0 | R | FALSE |
| 152 | 83 | CH | 4 | 4 | 4 | 4 | 0 | 0 | R | FALSE |
| 153 | 162 | CHX | 5 | 5 | 5 | 5 | 0 | 0 | R | TRUE |
| 154 | 56 | CH | 5 | 5 | 5 | 5 | 0 | 0 | R | FALSE |
| 155 | 74 | AAX | 2 | 2 | 2 | 2 | 0 | 0 | NR | TRUE |
| 156 | 22 | AAX | 2 | 2 | 2 | 2 | 0 | 0 | NR | TRUE |
| 157 | 245 | CH | 6 | 6 | 6 | 6 | 0 | 0 | R | FALSE |
| 158 | 328 | AAX | 7 | 7 | 7 | 7 | 0 | 0 | R | TRUE |
| 159 | 621 | AAX | 7 | 6 | 7 | 6 | 0 | 0 | R | TRUE |
| 160 | 156 | FR | 4 | 4 | 4 | 4 | 0 | 0 | R | TRUE |
| 161 | 43 | FRX | 5 | 5 | 5 | 5 | 0 | 0 | R | TRUE |
| 162 | 73 | FR | 5 | 5 | 5 | 5 | 0 | 0 | R | TRUE |
| 163 | 67 | FR | 4 | 4 | 4 | 4 | 0 | 0 | R | FALSE |
| 164 | 128 | FR | 7 | 7 | 7 | 7 | 0 | 0 | R | FALSE |
| 165 | 229 | AA | 4 | 4 | 8 | 10 | 4 | 6 | R | TRUE |
| 166 | 62 | HOL | 4 | 4 | 4 | 4 | 0 | 0 | R | TRUE |
| 167 | 36 | HOL | 3 | 3 | 3 | 3 | 0 | 0 | R | TRUE |
| 168 | 105 | HOL | 4 | 4 | 4 | 4 | 0 | 0 | R | FALSE |
| 169 | 88 | HOL | 4 | 4 | 4 | 4 | 0 | 0 | R | FALSE |
| 170 | 1784 | SIMX | 9 | 9 | 9 | 9 | 0 | 0 | R | TRUE |
| 171 | 538 | LIMX | 10 | 10 | 10 | 10 | 0 | 0 | R | TRUE |
| 172 | 297 | HOL | 4 | 4 | 4 | 4 | 0 | 0 | R | TRUE |
| 173 | 51 | HOL | 3 | 3 | 3 | 3 | 0 | 0 | R | TRUE |
| 174 | 362 | HOL | 5 | 5 | 5 | 5 | 0 | 0 | R | FALSE |
| 175 | 817 | HOL | 6 | 6 | 6 | 6 | 0 | 0 | R | TRUE |
| 176 | 785 | HOL | 7 | 7 | 7 | 7 | 0 | 0 | R | TRUE |
| 177 | 1153 | HOL | 8 | 8 | 8 | 8 | 0 | 0 | R | FALSE |
| 178 | 304 | HOL | 6 | 6 | 6 | 6 | 0 | 0 | R | FALSE |
| 179 | 753 | SIMX | 8 | 8 | 8 | 8 | 0 | 0 | R | TRUE |
| 180 | 1414 | AA | 7 | 7 | 7 | 7 | 0 | 0 | R | TRUE |
| 181 | 11 | AAX | 4 | 4 | 4 | 4 | 0 | 0 | R | FALSE |
| 182 | 38 | AAX | 4 | 4 | 4 | 4 | 0 | 0 | R | FALSE |
| 183 | 716 | HOL | 7 | 7 | 7 | 7 | 0 | 0 | R | TRUE |
| 184 | 497 | AAX | 6 | 6 | 6 | 6 | 0 | 0 | NR | TRUE |
| 185 | 281 | HOL | 6 | 6 | 6 | 6 | 0 | 0 | R | FALSE |
| 186 | 621 | HFD | 8 | 8 | 8 | 8 | 0 | 0 | R | TRUE |
| 187 | 250 | HOL | 8 | 8 | 8 | 8 | 0 | 0 | R | TRUE |
| 188 | 4 | AAX | 3 | 3 | 3 | 3 | 0 | 0 | R | TRUE |
| 189 | 57 | HFDX | 8 | 8 | 8 | 8 | 0 | 0 | R | FALSE |
| 190 | 12 | FR | 4 | 4 | 4 | 4 | 0 | 0 | R | TRUE |
| 191 | 1951 | HOL | 7 | 7 | 7 | 7 | 0 | 0 | R | FALSE |
| 192 | 1077 | CHX | 9 | 10 | 9 | 10 | 0 | 0 | R | TRUE |
| 193 | 474 | LIMX | 8 | 8 | 8 | 8 | 0 | 0 | R | TRUE |
| 194 | 146 | LIMX | 8 | 8 | 8 | 8 | 0 | 0 | R | FALSE |
| 195 | 1081 | LIMX | 10 | 10 | 10 | 10 | 0 | 0 | R | FALSE |
| 196 | 99 | LIMX | 7 | 7 | 7 | 7 | 0 | 0 | R | TRUE |
| 197 | 625 | CHX | 10 | 9 | 10 | 9 | 0 | 0 | R | FALSE |
| 198 | 164 | CHX | 3 | 4 | 3 | 4 | 0 | 0 | R | TRUE |
| 199 | 1215 | CHX | 12 | 12 | 12 | 12 | 0 | 0 | R | FALSE |
| 200 | 134 | CHX | 6 | 9 | 6 | 9 | 0 | 0 | R | FALSE |
| 201 | 147 | CH | 4 | 4 | 4 | 4 | 0 | 0 | R | TRUE |
| 202 | 306 | WB | 8 | 8 | 8 | 8 | 0 | 0 | NR | FALSE |
| 203 | 315 | WB | 8 | 8 | 8 | 8 | 0 | 0 | NR | FALSE |
| 204 | 155 | BBX | 8 | 8 | 14 | 11 | 6 | 3 | R | FALSE |
| 205 | 197 | LIMX | 8 | 8 | 8 | 8 | 0 | 0 | R | TRUE |
| 206 | 219 | BBX | 7 | 7 | 7 | 7 | 0 | 0 | R | FALSE |
| 207 | 212 | BBX | 11 | 11 | 11 | 11 | 0 | 0 | R | FALSE |
| 208 | 175 | LIMX | 8 | 8 | 8 | 8 | 0 | 0 | R | TRUE |
| 209 | 171 | LIMX | 8 | 8 | 8 | 8 | 0 | 0 | R | TRUE |
| 210 | 145 | AAX | 4 | 4 | 4 | 4 | 0 | 0 | R | TRUE |
| 211 | 350 | SIMX | 7 | 7 | 7 | 7 | 0 | 0 | R | FALSE |
| 212 | 682 | SIMX | 7 | 7 | 7 | 7 | 0 | 0 | R | FALSE |
| 213 | 741 | CHX | 13 | 13 | 13 | 13 | 0 | 0 | NR | FALSE |
| 214 | 1836 | BAX | 8 | 8 | 8 | 8 | 0 | 0 | R | FALSE |
| 215 | 1136 | LIMX | 8 | 8 | 8 | 8 | 0 | 0 | R | TRUE |
| 216 | 32 | CHX | 7 | 7 | 7 | 7 | 0 | 0 | R | TRUE |
| 217 | 99 | J | 4 | 4 | 4 | 4 | 0 | 0 | R | FALSE |
| 218 | 184 | CHX | 7 | 6 | 7 | 7 | 0 | 1 | NR | FALSE |
| 219 | 63 | HOL | 2 | 2 | 2 | 2 | 0 | 0 | R | FALSE |
| 220 | 954 | HOL | 5 | 5 | 11 | 11 | 6 | 6 | R | TRUE |
| 221 | 92 | HFDX | 4 | 4 | 4 | 4 | 0 | 0 | NR | TRUE |
| 222 | 51 | AA | 6 | 6 | 6 | 6 | 0 | 0 | R | TRUE |
| 223 | 11 | AA | 4 | 4 | 4 | 4 | 0 | 0 | R | TRUE |
| 224 | 11 | AA | 4 | 4 | 4 | 4 | 0 | 0 | R | TRUE |
| 225 | 19 | AA | 4 | 4 | 4 | 4 | 0 | 0 | R | TRUE |
| 226 | 17 | AA | 4 | 4 | 4 | 4 | 0 | 0 | R | TRUE |
| 227 | 16 | AA | 4 | 4 | 4 | 4 | 0 | 0 | R | TRUE |
| 228 | 76 | AA | 7 | 7 | 7 | 7 | 0 | 0 | R | TRUE |
| 229 | 30 | HOL | 4 | 4 | 4 | 4 | 0 | 0 | R | FALSE |
| 230 | 312 | HOL | 6 | 6 | 6 | 6 | 0 | 0 | R | TRUE |
| 231 | 52 | SIMX | 4 | 4 | 4 | 4 | 0 | 0 | NR | FALSE |
| 232 | 392 | HOL | 5 | 5 | 5 | 5 | 0 | 0 | NR | TRUE |
| 233 | 1471 | HOL | 6 | 6 | 6 | 6 | 0 | 0 | NR | TRUE |
| 234 | 898 | HOL | 7 | 7 | 7 | 7 | 0 | 0 | NR | TRUE |
| 235 | 942 | HOL | 7 | 7 | 7 | 7 | 0 | 0 | NR | FALSE |
| 236 | 453 | FR | 6 | 6 | 6 | 6 | 0 | 0 | NR | TRUE |
| 237 | 713 | FR | 5 | 5 | 5 | 5 | 0 | 0 | NR | FALSE |
| 238 | 329 | FRX | 5 | 5 | 5 | 5 | 0 | 0 | NR | TRUE |
| 239 | 150 | HOL | 5 | 5 | 5 | 5 | 0 | 0 | NR | FALSE |
| 240 | 327 | FRX | 6 | 6 | 6 | 6 | 0 | 0 | NR | FALSE |
| 241 | 306 | FRX | 5 | 5 | 5 | 5 | 0 | 0 | NR | FALSE |
| 242 | 235 | HOL | 5 | 5 | 5 | 5 | 0 | 0 | R | FALSE |
| 243 | 47 | HOL | 23 | 23 | 23 | 23 | 0 | 0 | R | FALSE |
| 244 | 296 | HFDX | 6 | 6 | 6 | 6 | 0 | 0 | NR | TRUE |
| 245 | 311 | FR | 5 | 5 | 5 | 5 | 0 | 0 | NR | TRUE |
| 246 | 885 | HFDX | 12 | 12 | 12 | 12 | 0 | 0 | NR | FALSE |
| 247 | 345 | HOL | 4 | 4 | 4 | 4 | 0 | 0 | R | FALSE |
| 248 | 35 | AAX | 5 | 5 | 5 | 5 | 0 | 0 | NR | TRUE |
| 249 | 43 | AAX | 2 | 2 | 2 | 2 | 0 | 0 | R | TRUE |
| 250 | 188 | AAX | 6 | 6 | 6 | 6 | 0 | 0 | R | FALSE |
| 251 | 165 | AAX | 6 | 6 | 6 | 6 | 0 | 0 | R | TRUE |
| 252 | 224 | AAX | 7 | 7 | 7 | 7 | 0 | 0 | NR | TRUE |
| 253 | 80 | AAX | 3 | 3 | 3 | 3 | 0 | 0 | NR | FALSE |
| 254 | 79 | AAX | 5 | 5 | 5 | 5 | 0 | 0 | R | TRUE |
| 255 | 266 | AAX | 7 | 7 | 7 | 7 | 0 | 0 | NR | TRUE |
| 256 | 75 | AAX | 2 | 2 | 2 | 2 | 0 | 0 | NR | FALSE |
| 257 | 176 | AAX | 6 | 6 | 6 | 6 | 0 | 0 | R | TRUE |
| 258 | 770 | HOL | 6 | 7 | 6 | 7 | 0 | 0 | R | FALSE |
| 259 | 125 | HOL | 3 | 3 | 3 | 3 | 0 | 0 | R | FALSE |
| 260 | 129 | AA | 4 | 4 | 4 | 4 | 0 | 0 | NR | TRUE |
| 261 | 1369 | LIMX | 10 | 10 | 10 | 10 | 0 | 0 | R | FALSE |
| 262 | 324 | SIMX | 6 | 6 | 6 | 6 | 0 | 0 | NR | TRUE |
| 263 | 513 | BBX | 6 | 6 | 6 | 6 | 0 | 0 | NR | FALSE |
| 264 | 217 | HOL | 4 | 4 | 4 | 4 | 0 | 0 | R | FALSE |
| 265 | 462 | BBX | 6 | 6 | 6 | 6 | 0 | 0 | NR | FALSE |
| 266 | 337 | HOL | 6 | 6 | 6 | 6 | 0 | 0 | R | TRUE |
| 267 | 231 | HOL | 4 | 4 | 4 | 4 | 0 | 0 | R | FALSE |
| 268 | 230 | HOL | 4 | 4 | 4 | 4 | 0 | 0 | R | TRUE |
| 269 | 53 | FRX | 4 | 4 | 4 | 4 | 0 | 0 | R | TRUE |
| 270 | 90 | FRX | 2 | 2 | 2 | 2 | 0 | 0 | R | TRUE |
| 271 | 83 | FRX | 3 | 3 | 3 | 3 | 0 | 0 | R | TRUE |
| 272 | 81 | FRX | 4 | 4 | 4 | 4 | 0 | 0 | R | TRUE |
| 273 | 169 | FRX | 4 | 4 | 4 | 4 | 0 | 0 | R | TRUE |
| 274 | 41 | FRX | 4 | 4 | 4 | 4 | 0 | 0 | R | TRUE |
| 275 | 447 | HFDX | 8 | 8 | 8 | 8 | 0 | 0 | R | FALSE |
| 276 | 76 | SIMX | 4 | 4 | 4 | 4 | 0 | 0 | R | FALSE |
| 277 | 46 | SIMX | 4 | 4 | 4 | 4 | 0 | 0 | R | TRUE |
| 278 | 365 | HOL | 5 | 5 | 5 | 5 | 0 | 0 | NR | FALSE |
| 279 | 275 | HOL | 4 | 4 | 4 | 4 | 0 | 0 | R | FALSE |
| 280 | 406 | HOL | 6 | 6 | 6 | 6 | 0 | 0 | R | TRUE |
| 281 | 19 | HOL | 4 | 4 | 4 | 4 | 0 | 0 | R | TRUE |
| 282 | 640 | HFDX | 8 | 8 | 8 | 8 | 0 | 0 | R | FALSE |
| 283 | 2846 | AAX | 8 | 8 | 8 | 8 | 0 | 0 | R | TRUE |
| 284 | 1748 | LIMX | 10 | 10 | 10 | 10 | 0 | 0 | R | TRUE |
| 285 | 1336 | AAX | 5 | 5 | 5 | 5 | 0 | 0 | R | TRUE |
| 286 | 202 | BBX | 5 | 5 | 5 | 5 | 0 | 0 | NR | TRUE |
| 287 | 1812 | HOL | 6 | 6 | 6 | 6 | 0 | 0 | NR | FALSE |
| 288 | 1498 | CHX | 8 | 8 | 8 | 8 | 0 | 0 | R | TRUE |
| 289 | 481 | CHX | 9 | 10 | 9 | 10 | 0 | 0 | R | TRUE |
| 290 | 1285 | AAX | 7 | 6 | 7 | 6 | 0 | 0 | R | TRUE |
| 291 | 160 | FR | 3 | 3 | 3 | 3 | 0 | 0 | R | TRUE |
| 292 | 184 | HOL | 4 | 4 | 4 | 4 | 0 | 0 | R | TRUE |
| 293 | 736 | HOL | 6 | 6 | 6 | 6 | 0 | 0 | R | FALSE |
| 294 | 108 | HOL | 2 | 2 | 2 | 2 | 0 | 0 | R | TRUE |
| 295 | 62 | HOLX | 4 | 4 | 4 | 4 | 0 | 0 | R | TRUE |
| 296 | 960 | FR | 5 | 5 | 5 | 5 | 0 | 0 | R | FALSE |
| 297 | 157 | FR | 4 | 4 | 4 | 4 | 0 | 0 | R | TRUE |
| 298 | 104 | HOL | 2 | 2 | 2 | 2 | 0 | 0 | R | TRUE |
| 299 | 344 | AAX | 10 | 10 | 10 | 10 | 0 | 0 | R | FALSE |
| 300 | 949 | HOL | 8 | 8 | 8 | 8 | 0 | 0 | NR | TRUE |
| 301 | 79 | AAX | 5 | 5 | 5 | 5 | 0 | 0 | NR | FALSE |
| 302 | 592 | CHX | 10 | 10 | 10 | 10 | 0 | 0 | R | TRUE |
| 303 | 524 | HOL | 7 | 8 | 7 | 8 | 0 | 0 | R | TRUE |
| 304 | 488 | FRX | 5 | 5 | 5 | 5 | 0 | 0 | R | TRUE |
| 305 | 560 | FRX | 5 | 5 | 5 | 5 | 0 | 0 | R | FALSE |
| 306 | 24 | LIMX | 4 | 4 | 4 | 4 | 0 | 0 | R | TRUE |
| 307 | 22 | LIMX | 4 | 4 | 4 | 4 | 0 | 0 | R | TRUE |
| 308 | 296 | AA | 7 | 7 | 7 | 7 | 0 | 0 | R | TRUE |
| 309 | 114 | HOL | 4 | 4 | 4 | 4 | 0 | 0 | R | FALSE |
| 310 | -130 | AA | 8 | 8 | 8 | 8 | 0 | 0 | NR | TRUE |
| 311 | -6 | BBX | 7 | 7 | 7 | 7 | 0 | 0 | NR | FALSE |
| 312 | 427 | DEX | 9 | 9 | 9 | 9 | 0 | 0 | R | FALSE |
| 313 | 114 | HFDX | 6 | 6 | 6 | 6 | 0 | 0 | R | TRUE |
| 314 | 492 | AAX | 6 | 6 | 6 | 6 | 0 | 0 | R | FALSE |
| 315 | 106 | HFDX | 6 | 6 | 6 | 6 | 0 | 0 | NR | FALSE |
| 316 | 2504 | HOL | 7 | 7 | 7 | 7 | 0 | 0 | R | FALSE |
| 317 | 286 | AAX | 6 | 6 | 6 | 6 | 0 | 0 | R | FALSE |
| 318 | 538 | FR | 8 | 8 | 8 | 8 | 0 | 0 | NR | TRUE |
| 319 | 602 | BBX | 6 | 6 | 6 | 6 | 0 | 0 | R | FALSE |
| 320 | 1750 | HOL | 8 | 8 | 10 | 8 | 2 | 0 | NR | TRUE |
| 321 | 394 | HOL | 7 | 7 | 7 | 7 | 0 | 0 | NR | FALSE |
| 322 | 599 | HOL | 8 | 8 | 8 | 8 | 0 | 0 | NR | FALSE |
| 323 | 55 | HOL | 4 | 4 | 4 | 4 | 0 | 0 | NR | TRUE |
| 324 | 2457 | AA | 12 | 12 | 12 | 12 | 0 | 0 | NR | TRUE |
| 325 | 2454 | AA | 12 | 12 | 12 | 12 | 0 | 0 | NR | TRUE |
| 326 | 284 | CHX | 8 | 8 | 8 | 8 | 0 | 0 | R | FALSE |
| 327 | 65 | AAX | 5 | 5 | 5 | 5 | 0 | 0 | R | FALSE |
| 328 | 689 | SIMX | 8 | 9 | 8 | 9 | 0 | 0 | NR | TRUE |
| 329 | 324 | AAX | 10 | 10 | 10 | 10 | 0 | 0 | R | FALSE |
| 330 | 1081 | HOL | 5 | 5 | 5 | 5 | 0 | 0 | NR | FALSE |
| 331 | 1707 | HOL | 5 | 5 | 7 | 7 | 2 | 2 | NR | TRUE |
| 332 | 1116 | SIMX | 10 | 10 | 16 | 14 | 6 | 4 | R | FALSE |
| 333 | 1323 | AA | 12 | 12 | 15 | 12 | 3 | 0 | NR | FALSE |
| 334 | 1326 | AA | 12 | 12 | 12 | 12 | 0 | 0 | NR | TRUE |
| 335 | 1286 | AAX | 12 | 12 | 18 | 12 | 6 | 0 | NR | FALSE |
| 336 | 1011 | AA | 12 | 12 | 22 | 16 | 10 | 4 | NR | FALSE |
| 337 | 1331 | AA | 12 | 12 | 12 | 12 | 0 | 0 | NR | TRUE |
| 338 | 1180 | HOL | 6 | 6 | 6 | 6 | 0 | 0 | NR | TRUE |
| 339 | 1478 | HOL | 6 | 6 | 6 | 6 | 0 | 0 | NR | TRUE |
| 340 | 188 | LIM | 5 | 5 | 5 | 5 | 0 | 0 | NR | TRUE |
| 341 | 185 | LIM | 4 | 4 | 4 | 4 | 0 | 0 | NR | FALSE |
| 342 | 1190 | FR | 8 | 8 | 8 | 8 | 0 | 0 | NR | TRUE |
| 343 | 325 | LIMX | 10 | 10 | 10 | 10 | 0 | 0 | R | TRUE |
| 344 | 92 | HFDX | 6 | 6 | 6 | 6 | 0 | 0 | NR | FALSE |
| 345 | 190 | HOL | 5 | 5 | 5 | 5 | 0 | 0 | NR | FALSE |
| 346 | 837 | HFDX | 10 | 10 | 10 | 10 | 0 | 0 | NR | TRUE |
| 347 | 380 | AA | 10 | 10 | 10 | 10 | 0 | 0 | R | FALSE |
| 348 | 51 | CHX | 4 | 4 | 4 | 4 | 0 | 0 | NR | TRUE |
| 349 | 75 | CHX | 9 | 9 | 9 | 9 | 0 | 0 | R | FALSE |
| 350 | 447 | SIMX | 10 | 8 | 10 | 8 | 0 | 0 | R | FALSE |
| 351 | 1652 | DEV | 12 | 12 | 12 | 12 | 0 | 0 | NR | TRUE |
| 352 | 243 | SDEV | 9 | 9 | 9 | 9 | 0 | 0 | R | TRUE |
| 353 | 1260 | SDEV | 10 | 10 | 10 | 10 | 0 | 0 | R | FALSE |
| 354 | 139 | SDEV | 9 | 8 | 9 | 8 | 0 | 0 | R | TRUE |
| 355 | 2225 | SDEV | 10 | 11 | 10 | 11 | 0 | 0 | R | TRUE |
| 356 | 514 | SDEV | 10 | 12 | 13 | 12 | 3 | 0 | R | TRUE |
| 357 | 241 | LIMX | 3 | 3 | 3 | 3 | 0 | 0 | NR | TRUE |
| 358 | 2564 | DEX | 7 | 7 | 7 | 7 | 0 | 0 | R | FALSE |
| 359 | 891 | LIMX | 8 | 8 | 8 | 8 | 0 | 0 | R | TRUE |
| 360 | 383 | AAX | 4 | 4 | 4 | 4 | 0 | 0 | NR | TRUE |
| 361 | 122 | LIMX | 6 | 6 | 6 | 6 | 0 | 0 | NR | TRUE |
| 362 | 1249 | SIMX | 8 | 8 | 8 | 8 | 0 | 0 | NR | TRUE |
| 363 | -116 | SIMX | 8 | 8 | 8 | 8 | 0 | 0 | NR | FALSE |
| 364 | 42 | J | 5 | 5 | 5 | 5 | 0 | 0 | NR | FALSE |
| 365 | 380 | BBX | 7 | 7 | 7 | 7 | 0 | 0 | NR | TRUE |
| 366 | 2081 | HOL | 8 | 8 | 11 | 8 | 3 | 0 | R | TRUE |
| 367 | 555 | LIMX | 6 | 6 | 6 | 6 | 0 | 0 | R | FALSE |
| 368 | 365 | DEV | 9 | 10 | 9 | 10 | 0 | 0 | NR | FALSE |
| 369 | 160 | BBX | 5 | 5 | 5 | 5 | 0 | 0 | R | TRUE |
| 370 | 1258 | LIMX | 8 | 8 | 8 | 8 | 0 | 0 | NR | TRUE |
| 371 | 140 | LIMX | 7 | 7 | 7 | 7 | 0 | 0 | R | TRUE |
| 372 | 309 | WB | 12 | 12 | 12 | 12 | 0 | 0 | NR | FALSE |
| 373 | 307 | WB | 12 | 12 | 12 | 12 | 0 | 0 | NR | FALSE |
| 374 | 784 | LIMX | 8 | 8 | 14 | 18 | 6 | 10 | IR | TRUE |
| 375 | 315 | CHX | 7 | 7 | 7 | 7 | 0 | 0 | NR | TRUE |
| 376 | 3538 | LIMX | 5 | 5 | 5 | 11 | 0 | 6 | R | TRUE |
| 377 | 272 | AA | 7 | 8 | 12 | 14 | 5 | 6 | R | TRUE |
| 378 | 69 | LIMX | 5 | 5 | 5 | 5 | 0 | 0 | NR | TRUE |
| 379 | 2084 | LIMX | 7 | 7 | 7 | 7 | 0 | 0 | R | TRUE |
| 380 | 1110 | FR | 5 | 5 | 5 | 5 | 0 | 0 | NR | FALSE |
| 381 | 135 | HFD | 9 | 8 | 9 | 8 | 0 | 0 | R | FALSE |
| 382 | 135 | HFD | 8 | 9 | 8 | 9 | 0 | 0 | R | FALSE |
| 383 | 153 | HFD | 9 | 9 | 9 | 9 | 0 | 0 | R | TRUE |
| 384 | 156 | HFD | 11 | 11 | 11 | 11 | 0 | 0 | R | FALSE |
| 385 | 155 | HFD | 8 | 9 | 8 | 9 | 0 | 0 | R | FALSE |
| 386 | 772 | BAX | 6 | 6 | 6 | 6 | 0 | 0 | NR | TRUE |
| 387 | 928 | WB | 8 | 8 | 8 | 8 | 0 | 0 | NR | FALSE |
| 388 | 1421 | LIMX | 7 | 7 | 7 | 7 | 0 | 0 | R | FALSE |
| 389 | 284 | HFD | 6 | 6 | 6 | 6 | 0 | 0 | NR | FALSE |
| 390 | 357 | HFD | 6 | 6 | 6 | 6 | 0 | 0 | NR | FALSE |
| 391 | 253 | WB | 7 | 7 | 7 | 7 | 0 | 0 | NR | FALSE |
| 392 | 814 | HFDX | 8 | 8 | 8 | 8 | 0 | 0 | NR | FALSE |
| 393 | 564 | CHX | 8 | 8 | 8 | 8 | 0 | 0 | NR | TRUE |
| 394 | 578 | CHX | 7 | 7 | 7 | 7 | 0 | 0 | NR | TRUE |
| 395 | 574 | CHX | 9 | 9 | 14 | 9 | 5 | 0 | NR | FALSE |
| 396 | 577 | CHX | 9 | 9 | 9 | 9 | 0 | 0 | NR | TRUE |
| 397 | 567 | CHX | 8 | 8 | 8 | 8 | 0 | 0 | NR | TRUE |
| 398 | 567 | CHX | 9 | 9 | 17 | 12 | 8 | 3 | NR | FALSE |
| 399 | 575 | CHX | 8 | 8 | 8 | 8 | 0 | 0 | NR | TRUE |
| 400 | 85 | LIM | 8 | 8 | 8 | 8 | 0 | 0 | NR | TRUE |
| 401 | 44 | LIMX | 6 | 6 | 6 | 6 | 0 | 0 | NR | TRUE |
| 402 | 591 | SIMX | 8 | 8 | 8 | 8 | 0 | 0 | NR | TRUE |
| 403 | 537 | SIMX | 10 | 10 | 10 | 10 | 0 | 0 | NR | FALSE |
| 404 | 539 | SIMX | 10 | 10 | 10 | 10 | 0 | 0 | NR | TRUE |
| 405 | 296 | LIMX | 8 | 8 | 8 | 8 | 0 | 0 | NR | TRUE |
| 406 | 82 | HOL | 5 | 5 | 5 | 5 | 0 | 0 | NR | FALSE |
| 407 | 322 | AA | 6 | 6 | 6 | 6 | 0 | 0 | NR | TRUE |
| 408 | 405 | LIMX | 8 | 8 | 8 | 8 | 0 | 0 | NR | FALSE |
| 409 | 371 | AA | 7 | 7 | 7 | 7 | 0 | 0 | NR | TRUE |
| 410 | 126 | CHX | 10 | 10 | 10 | 10 | 0 | 0 | NR | TRUE |
| 411 | 361 | LIMX | 7 | 7 | 9 | 8 | 2 | 1 | NR | TRUE |
| 412 | 48 | SIMX | 5 | 5 | 5 | 5 | 0 | 0 | NR | TRUE |
| 413 | 734 | SIMX | 6 | 6 | 6 | 6 | 0 | 0 | NR | TRUE |
| 414 | 82 | BBX | 12 | 12 | 12 | 12 | 0 | 0 | NR | TRUE |
| 415 | 137 | AAX | 5 | 5 | 5 | 5 | 0 | 0 | NR | FALSE |
| 416 | 15 | LIM | 3 | 3 | 3 | 3 | 0 | 0 | NR | FALSE |
| 417 | 208 | SDEV | 6 | 6 | 6 | 6 | 0 | 0 | NR | TRUE |
| 418 | 232 | SDEV | 6 | 6 | 6 | 6 | 0 | 0 | NR | TRUE |
| 419 | 229 | WB | 9 | 9 | 9 | 9 | 0 | 0 | NR | FALSE |
| 420 | 301 | SIMX | 4 | 4 | 4 | 4 | 0 | 0 | NR | TRUE |
| 421 | 257 | LIMX | 3 | 3 | 3 | 3 | 0 | 0 | NR | FALSE |
| 422 | 190 | LIMX | 2 | 2 | 2 | 2 | 0 | 0 | NR | FALSE |
| 423 | 200 | LIMX | 3 | 3 | 3 | 3 | 0 | 0 | NR | FALSE |
| 424 | 280 | LIMX | 2 | 2 | 2 | 2 | 0 | 0 | NR | TRUE |
| 425 | 116 | FRX | 4 | 4 | 4 | 4 | 0 | 0 | NR | TRUE |
| 426 | 162 | LIMX | 3 | 3 | 3 | 3 | 0 | 0 | NR | FALSE |
| 427 | 109 | LIMX | 4 | 4 | 4 | 4 | 0 | 0 | NR | TRUE |
| 428 | 267 | FRX | 2 | 2 | 2 | 2 | 0 | 0 | NR | FALSE |
| 429 | 119 | LIMX | 2 | 2 | 2 | 2 | 0 | 0 | NR | TRUE |
| 430 | 108 | LIMX | 9 | 9 | 9 | 9 | 0 | 0 | NR | TRUE |
| 431 | 142 | HOL | 4 | 4 | 4 | 4 | 0 | 0 | NR | FALSE |
| 432 | 138 | LIM | 3 | 3 | 3 | 3 | 0 | 0 | NR | TRUE |
| 433 | 137 | LIMX | 3 | 3 | 3 | 3 | 0 | 0 | NR | TRUE |
| 434 | 53 | LIMX | 8 | 8 | 8 | 8 | 0 | 0 | NR | TRUE |
| 435 | 76 | LIMX | 8 | 8 | 8 | 8 | 0 | 0 | NR | TRUE |
| 436 | 106 | LIMX | 7 | 8 | 7 | 8 | 0 | 0 | NR | FALSE |
| 437 | 83 | LIMX | 5 | 5 | 5 | 5 | 0 | 0 | NR | TRUE |
| 438 | 142 | LIMX | 3 | 3 | 3 | 3 | 0 | 0 | NR | FALSE |
| 439 | 112 | HOL | 3 | 2 | 3 | 2 | 0 | 0 | IR | TRUE |
| 440 | 13 | HFDX | 6 | 6 | 6 | 6 | 0 | 0 | R | FALSE |
| 441 | 295 | LIMX | 9 | 9 | 9 | 9 | 0 | 0 | NR | TRUE |
| 442 | 95 | CH | 4 | 4 | 4 | 4 | 0 | 0 | NR | TRUE |
| 443 | 385 | LIMX | 5 | 5 | 5 | 5 | 0 | 0 | NR | TRUE |
| 444 | 290 | LIMX | 8 | 8 | 8 | 8 | 0 | 0 | NR | TRUE |
| 445 | 320 | LIMX | 7 | 7 | 13 | 12 | 6 | 5 | NR | TRUE |
| 446 | 351 | BBX | 10 | 10 | 10 | 10 | 0 | 0 | R | FALSE |
| 447 | 251 | CHX | 6 | 6 | 6 | 6 | 0 | 0 | NR | TRUE |
| 448 | 270 | LIMX | 6 | 6 | 6 | 6 | 0 | 0 | NR | TRUE |
| 449 | 64 | WB | 8 | 8 | 8 | 8 | 0 | 0 | NR | FALSE |
| 450 | 276 | HFD | 6 | 6 | 6 | 6 | 0 | 0 | NR | FALSE |
| 451 | 55 | BBX | 6 | 9 | 6 | 9 | 0 | 0 | NR | FALSE |
| 452 | 346 | LIMX | 6 | 6 | 6 | 6 | 0 | 0 | NR | TRUE |
| 453 | 353 | LIMX | 8 | 8 | 8 | 8 | 0 | 0 | NR | FALSE |
| 454 | 316 | BAX | 7 | 7 | 7 | 7 | 0 | 0 | NR | TRUE |
| 455 | 550 | LIMX | 8 | 8 | 8 | 8 | 0 | 0 | NR | FALSE |
| 456 | 543 | LIMX | 10 | 10 | 10 | 10 | 0 | 0 | NR | FALSE |
| 457 | 563 | CHX | 9 | 9 | 9 | 9 | 0 | 0 | NR | TRUE |
| 458 | 140 | HFDX | 6 | 6 | 6 | 6 | 0 | 0 | IR | TRUE |
| 459 | 2252 | HOL | 7 | 8 | 7 | 8 | 0 | 0 | NR | FALSE |
| 460 | 979 | BBX | 5 | 5 | 5 | 5 | 0 | 0 | R | FALSE |
| 461 | 837 | SIMX | 8 | 8 | 8 | 8 | 0 | 0 | NR | FALSE |
| 462 | 2438 | BBX | 10 | 9 | 10 | 9 | 0 | 0 | NR | TRUE |
| 463 | 289 | BBX | 5 | 5 | 5 | 5 | 0 | 0 | R | TRUE |
| 464 | 312 | FR | 5 | 5 | 5 | 5 | 0 | 0 | NR | TRUE |
| 465 | 127 | LIMX | 6 | 6 | 6 | 6 | 0 | 0 | R | TRUE |
| 466 | 1274 | DEV | 9 | 9 | 9 | 9 | 0 | 0 | R | FALSE |
| 467 | 86 | FR | 3 | 3 | 3 | 3 | 0 | 0 | NR | FALSE |
| 468 | 185 | FR | 5 | 5 | 5 | 5 | 0 | 0 | NR | FALSE |
| 469 | 110 | FR | 3 | 3 | 3 | 3 | 0 | 0 | NR | TRUE |
| 470 | 174 | FR | 4 | 4 | 4 | 4 | 0 | 0 | NR | FALSE |
| 471 | 115 | HFDX | 4 | 4 | 4 | 4 | 0 | 0 | NR | TRUE |
| 472 | 334 | FRX | 5 | 5 | 5 | 5 | 0 | 0 | NR | TRUE |
| 473 | 332 | FRX | 5 | 5 | 5 | 5 | 0 | 0 | NR | FALSE |
| 474 | 332 | FRX | 5 | 5 | 5 | 5 | 0 | 0 | NR | FALSE |
| 475 | 330 | FRX | 5 | 5 | 5 | 5 | 0 | 0 | NR | TRUE |
| 476 | 690 | HOL | 7 | 7 | 7 | 7 | 0 | 0 | NR | FALSE |
| 477 | 147 | HFDX | 6 | 6 | 6 | 6 | 0 | 0 | R | TRUE |
| 478 | 535 | HOLX | 7 | 7 | 7 | 7 | 0 | 0 | NR | TRUE |
| 479 | 877 | BBX | 7 | 7 | 7 | 7 | 0 | 0 | NR | TRUE |
| 480 | 2118 | FR | 5 | 6 | 11 | 11 | 6 | 5 | R | FALSE |
| 481 | 3190 | LIM | 6 | 6 | 6 | 6 | 0 | 0 | NR | FALSE |
| 482 | 2011 | HOLX | 8 | 8 | 8 | 8 | 0 | 0 | R | FALSE |
| 483 | 2909 | HFDX | 6 | 6 | 6 | 6 | 0 | 0 | R | FALSE |
| 484 | 2186 | HOL | 5 | 5 | 5 | 5 | 0 | 0 | R | FALSE |
| 485 | 2803 | BAX | 8 | 8 | 8 | 8 | 0 | 0 | R | FALSE |
| 486 | 2772 | BAX | 10 | 10 | 10 | 10 | 0 | 0 | R | FALSE |
| 487 | 3540 | HFDX | 7 | 7 | 7 | 7 | 0 | 0 | NR | TRUE |
| 488 | 3223 | FR | 6 | 6 | 6 | 6 | 0 | 0 | NR | TRUE |
| 489 | 2568 | HOLX | 5 | 5 | 5 | 5 | 0 | 0 | NR | TRUE |
| 490 | 1512 | HOL | 6 | 6 | 6 | 6 | 0 | 0 | R | FALSE |
| 491 | 2762 | FR | 7 | 7 | 7 | 7 | 0 | 0 | NR | TRUE |
| 492 | 1789 | AA | 5 | 5 | 5 | 5 | 0 | 0 | R | TRUE |
| 493 | 2486 | DEX | 7 | 7 | 7 | 7 | 0 | 0 | R | FALSE |
| 494 | 3590 | HFDX | 6 | 6 | 6 | 6 | 0 | 0 | NR | FALSE |
| 495 | 2485 | CH | 8 | 8 | 8 | 10 | 0 | 2 | R | FALSE |
| 496 | 2695 | FR | 6 | 6 | 6 | 6 | 0 | 0 | R | TRUE |
| 497 | 2660 | FR | 6 | 6 | 6 | 6 | 0 | 0 | R | TRUE |
| 498 | 2656 | FR | 6 | 6 | 6 | 6 | 0 | 0 | R | FALSE |
| 499 | 2397 | FR | 6 | 6 | 6 | 6 | 0 | 0 | R | TRUE |
| 500 | 2250 | FR | 6 | 6 | 6 | 6 | 0 | 0 | R | TRUE |
| 501 | 2168 | FR | 6 | 6 | 6 | 6 | 0 | 0 | R | TRUE |
| 502 | 1637 | FR | 6 | 6 | 6 | 6 | 0 | 0 | R | TRUE |
| 503 | 1625 | FR | 6 | 6 | 6 | 6 | 0 | 0 | R | TRUE |
| 504 | 2446 | SIMX | 10 | 8 | 10 | 8 | 0 | 0 | R | TRUE |
| 505 | 2772 | HOL | 4 | 5 | 4 | 5 | 0 | 0 | NR | FALSE |
| 506 | 4512 | HFD | 7 | 7 | 7 | 7 | 0 | 0 | R | TRUE |
| 507 | 2087 | WB | 8 | 8 | 8 | 8 | 0 | 0 | NR | FALSE |
| 508 | 2904 | FR | 7 | 7 | 11 | 10 | 4 | 3 | NR | TRUE |
| 509 | 2770 | CHX | 11 | 11 | 14 | 12 | 3 | 1 | NR | TRUE |
| 510 | 2297 | FR | 5 | 5 | 5 | 5 | 0 | 0 | NR | TRUE |
| 511 | 3654 | CHX | 8 | 8 | 8 | 8 | 0 | 0 | NR | FALSE |
| 512 | 3395 | DEV | 12 | 12 | 12 | 12 | 0 | 0 | R | TRUE |
| 513 | 2675 | SDEV | 10 | 10 | 10 | 10 | 0 | 0 | R | FALSE |
| 514 | 2346 | SDEV | 10 | 10 | 10 | 10 | 0 | 0 | R | TRUE |
| 515 | 2066 | FRX | 5 | 6 | 12 | 8 | 7 | 2 | R | FALSE |
| 516 | 1928 | HOL | 7 | 7 | 7 | 7 | 0 | 0 | NR | TRUE |
| 517 | 4133 | FR | 7 | 7 | 7 | 7 | 0 | 0 | R | FALSE |
| 518 | 2348 | FR | 8 | 8 | 8 | 8 | 0 | 0 | R | TRUE |
| 519 | 2574 | FR | 4 | 4 | 4 | 4 | 0 | 0 | NR | FALSE |
| 520 | 3076 | FR | 8 | 8 | 8 | 8 | 0 | 0 | NR | TRUE |
| 521 | 3045 | FR | 8 | 8 | 8 | 8 | 0 | 0 | NR | TRUE |
| 522 | 2674 | FR | 8 | 8 | 8 | 8 | 0 | 0 | NR | TRUE |
| 523 | 2664 | FR | 8 | 8 | 8 | 8 | 0 | 0 | NR | TRUE |
| 524 | 2382 | FR | 8 | 8 | 8 | 8 | 0 | 0 | NR | TRUE |
| 525 | 2374 | HOL | 5 | 5 | 5 | 5 | 0 | 0 | NR | TRUE |
| 526 | 2727 | FR | 7 | 7 | 7 | 7 | 0 | 0 | NR | TRUE |
